# Solar‐Powered Electrokinetic Filtration using Hierarchical Porous Membranes for the Off‐Grid Removal of Ultrafine Contaminants

**DOI:** 10.1002/advs.202515435

**Published:** 2025-10-14

**Authors:** Woonjae Choi, Minsoo Lee, Young June Park, Seungbin Yoon, Geunbae Lim

**Affiliations:** ^1^ Department of Mechanical Engineering Pohang University of Science and Technology (POSTECH) 77 Cheongam‐Ro, Nam‐Gu Pohang Gyeongbuk 37673 Republic of Korea

**Keywords:** electrokinetics, hierarchical structures, ion concentration polarization, nanoparticles, porous materials

## Abstract

The removal of ultrafine suspended solids from water remains a critical challenge due to the tradeoffs among permeability, energy consumption, and fouling resistance. Electrokinetic techniques offer a membrane‐free alternative; however, they are limited by low throughput, heavy reliance on auxiliary equipment, and technically demanding operations. Herein, a scalable, solar‐powered nanoelectrokinetic filtration platform is proposed, which enables the efficient removal of nanoparticles (NPs), organic dyes, and *Escherichia coli (E. coli)* using micrometer‐scale porous membranes. The core component is a hierarchically structured membrane, consisting of a biodegradable cellulose–cotton scaffold coated with ion‐exchange resin and Nafion, forming confined microchannels and ion‐selective nanochannels that generate stable electrokinetic repulsion under an applied electric field. Powered by a photovoltaic module and operated under gravity‐driven flow (<1 kPa), the system achieves >99.9% removal of sub‐50‐nm particles, including species <10 nm, at high fluxes (>400 L m^−2^ h^−1^), and efficiently rejects gold NPs, dyes, and *E. coli*, with stable voltage and energy consumption (≈10 Wh L^−1^) under tap water conditions. Moreover, the antifouling washable membrane retains its performance over 20 reuse cycles and supports linear scalability. Overall, the present study establishes a new class of electrokinetic‐assisted filtration systems with high potential for off‐grid water purification in resource‐limited environments.

## Graphical abstract

A solar‐powered nanoelectrokinetic filtration system enables high‐flux removal of ultrafine contaminants without relying on nanoscale pores or pressure‐driven membranes, offering a sustainable solution for the off‐grid water purification.

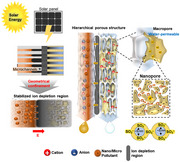

## 1 Introduction

Access to safe drinking water continues to improve; however, one in four people globally still experiences severe water scarcity, and more than 3.5 billion are projected to face water stress by 2025.^[^ ^1^, ^2^, ^3^, ^4^ ^]^ These statistics highlight the urgent requirement to bolster efforts to achieve Sustainable Development Goal 6,^[^ ^5^, ^6^ ^]^ which targets universal access to safe drinking water. Conventional water purification processes involve multiple stages, including coagulation, sedimentation, filtration, disinfection, and desalination. However, global water scarcity, which has been exacerbated by climate change and increasing industrial and agricultural demands, has necessitated the development of efficient and easy‐to‐use water treatment systems, especially in resource‐limited areas.

Although the removal of contaminants, such as dissolved ions, pathogens, and organic pollutants, remains important, the removal of suspended solids (SS) has attracted growing attention owing to their ubiquity, health implications, and the operational burden they impose on filtration systems.^[^ ^7^, ^8^, ^9^ ^]^ Moreover, ultrafine particles such as nanoplastics can accumulate in the human body and induce cellular damage, thereby posing serious health risks if not removed effectively.^[^ ^10^, ^11^, ^12^, ^13^, ^14^ ^]^ In many underdeveloped regions, the consumption of SS‐contaminated water remains an issue owing to limited awareness and poor access to adequate treatment infrastructure. Consequently, there is an urgent need for water treatment technologies that are not only effective but also simple, scalable, and well‐suited for deployment in resource‐limited settings.^[^ ^15^, ^16^, ^17^, ^18^ ^]^

**Figure 1:**
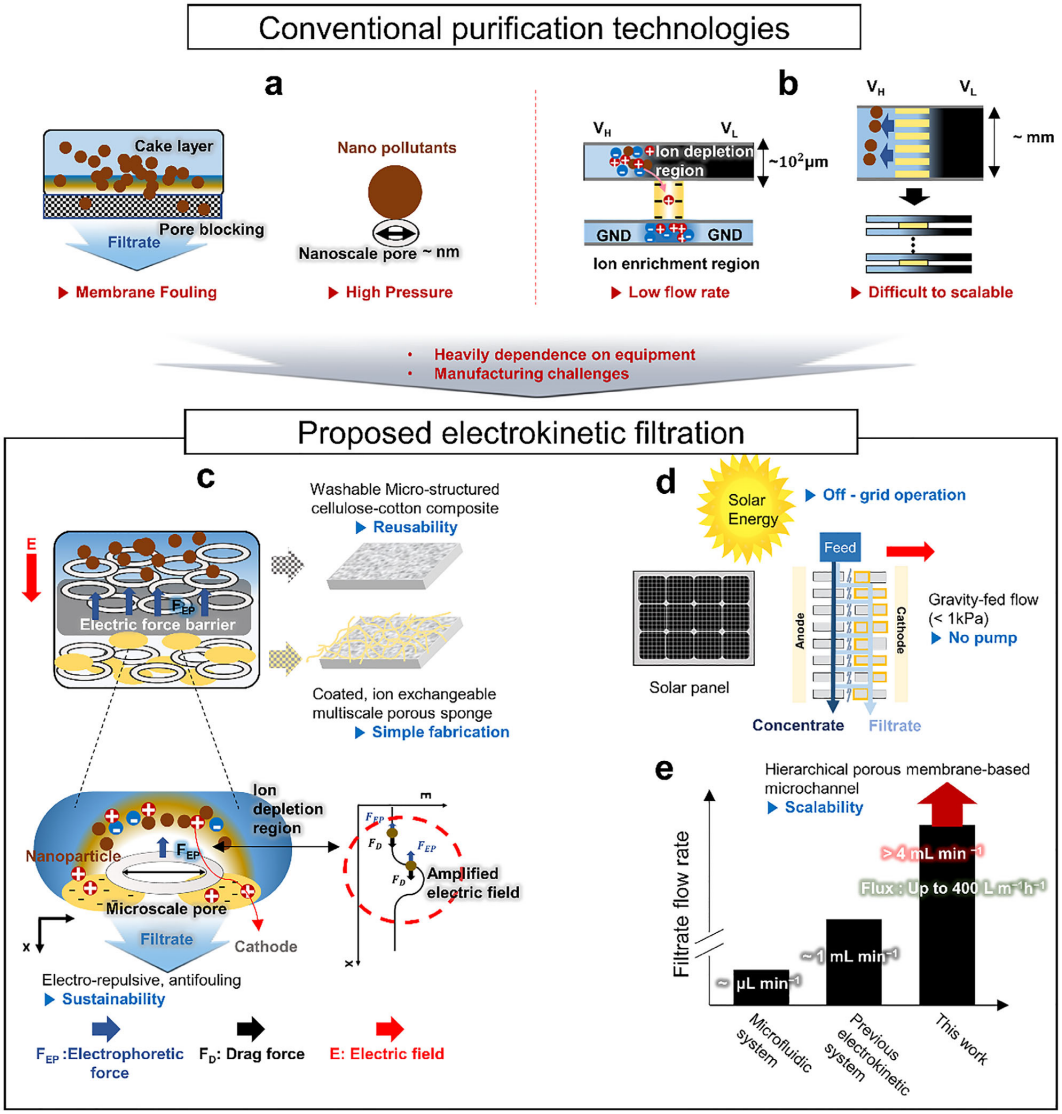
Schematic representations of (a) conventional membrane‐based filtration, b) the nanoelectrokinetic system, and c) the proposed scalable nanoelectrokinetic filtration platform using a washable, microstructured cellulose‐cotton composite coated with an ion‐exchangeable porous sponge. The applied electric field forms an ion depletion region that amplifies electrophoretic forces to repel charged nanoparticles against drag force. d) The system operates using solar energy and utilizes gravity‐driven flow (<1 kPa), allowing for off‐grid operation without the need for a pump. e) Comparison of filtrate flow rates from a conventional microfluidic systems, a previous electrokinetic system, and this study highlights the scalability achieved by the hierarchical porous membrane design.

To date, various methods, such as electrophoresis, centrifugation, and magnetic flocculation have been explored for the separation of ultrafine particles.^[^ ^19^, ^20^, ^21^, ^22^ ^]^ Among them, membrane filtration has been used extensively to remove SS, such as microplastics, microorganisms, and bacteria via size‐exclusion mechanisms.^[^ ^23^, ^24^, ^25^, ^26^ ^]^ However, as the target contaminants approach the nanoscale, the membrane pore sizes must be reduced accordingly, which significantly lowers the permeability and increases the risk of membrane fouling.^[^ ^27^, ^28^, ^29^, ^30^ ^]^ Moreover, the production of such membranes often requires advanced fabrication techniques, resulting in higher material costs and limited accessibility in resource‐constrained settings (**Figure** **1**a).

To overcome the limitations of size‐based membrane filtration, emerging microfluidic water treatment technologies^[^ ^31^, ^32^, ^33^, ^34^, ^35^ ^]^ that leverage nanofluidic electrokinetic phenomena are gaining increasing attention. Such systems can separate ultrafine particles measuring as small as 100 nm without the requirement for membranes;^[^ ^36^, ^37^ ^]^ When a direct current (DC) electric field is applied, counter‐ions migrate through the membrane, while co‐ions are excluded. This selective transport causes a concentration imbalance near the membrane interface, forming an ion depletion region characterized by a steep drop in ionic strength and increased local resistance, which in turn enhances the local electric field. The resulting field acts as a repulsive electrokinetic barrier to co‐ions and negatively charged species, effectively inhibiting their passage. However, their flow rates remain extremely low (≈µL min^−1^), and the fabrication processes are often complex and labor‐intensive (Figure 1b). Additionally, although parallelized designs can partially increase throughput, they introduce additional system complexity and require external tubing and pumps. To address such limitations, our group previously introduced a multiscale porous ion‐exchange membrane that enables stable and scalable electrokinetic operation.^[^ ^38^, ^39^, ^40^, ^41^ ^]^ In a previous study targeting plastic particle removal, effective separation required relatively high operating voltages (>100 V).^[^ ^39^ ^]^ Despite their promise, concerns persist about long‐term operation, particularly due to the risk of fouling and the limited understanding of performance stability over time. In addition, non‐uniform electric fields during scale‐up continue to hinder consistent filtration performance. Consequently, most electrokinetic filtration systems remain confined to laboratory‐scale applications owing to their heavy reliance on external power sources and supporting equipment.

The present study introduces a scalable electrokinetic filtration platform that combines high‐throughput operation, facile fabrication, and compatibility with solar‐powered systems (Figure 1c). This device features a hierarchical microporous structure composed of washable, biodegradable, and plant‐derived textiles (cellulose sponge cloth and cotton) integrated with a multiscale porous cation‐exchange membrane (MP‐CEM) conformally coated with a cation‐exchange resin. Upon the application of an electric field, this hierarchical architecture forms micro‐ and nanochannel‐like pathways, establishing a stable ion‐depletion zone with locally amplified electric fields. This enables electrokinetic rejection of sub‐50‐nm particulates while maintaining gravity‐driven bulk flow under a modest pressure drop. As a result, the system operates without high‐pressure pumps, mitigates membrane fouling, and offers long‐term reusability compared with conventional size‐exclusion membranes. To assess its suitability for off‐grid application, the filtration module is operated using power supplied from a solar‐charged battery system (Figure 1d). In addition, the membrane‐based microchannel design supports straightforward and linear scaling to larger areas, which has rarely been achieved in previously reported electrokinetic systems (Figure 1e). The approach above represents a compelling alternative to conventional filtration technologies considering its high separation efficiency, large flux, user‐friendly operation, long‐term durability, and low‐cost scalable manufacturing enabled by a hydrophilic hierarchical scaffold design. Notably, the system operates on solar power and requires neither pumps nor external energy sources, thereby minimizing its dependency on equipment. These features render it highly suitable for off‐grid, rural, and low‐resource settings where infrastructure is limited. Collectively, this work presents a practical and sustainable approach for producing safe drinking water, with the potential to replace conventional, energy‐intensive filtration systems.

## 2 Results and Discussion

### 2.1 Hierarchical Porous Membranes

The filtration system was designed for ease of fabrication using readily available commercial materials. The key components included a biodegradable, hydrophilic scaffold composed of cellulose and cotton sponge cloth, as well as a commercial cation‐exchange resin solution. The porous scaffold simultaneously functions as a fluidic channel and was selected because of its hydrophilicity, durability, porosity, 100% biodegradability, reusability, and plastic‐free composition (Figure S1, Supporting Information). The uncoated channel serves as the concentrate outlet, where accumulated particles are discharged. For this reason, cleanability and regenerability are important. Furthermore, it can be readily disposed of and replaced with low‐cost sponges without environmental concerns when the scaffold becomes worn or fouled.

**Figure 2:**
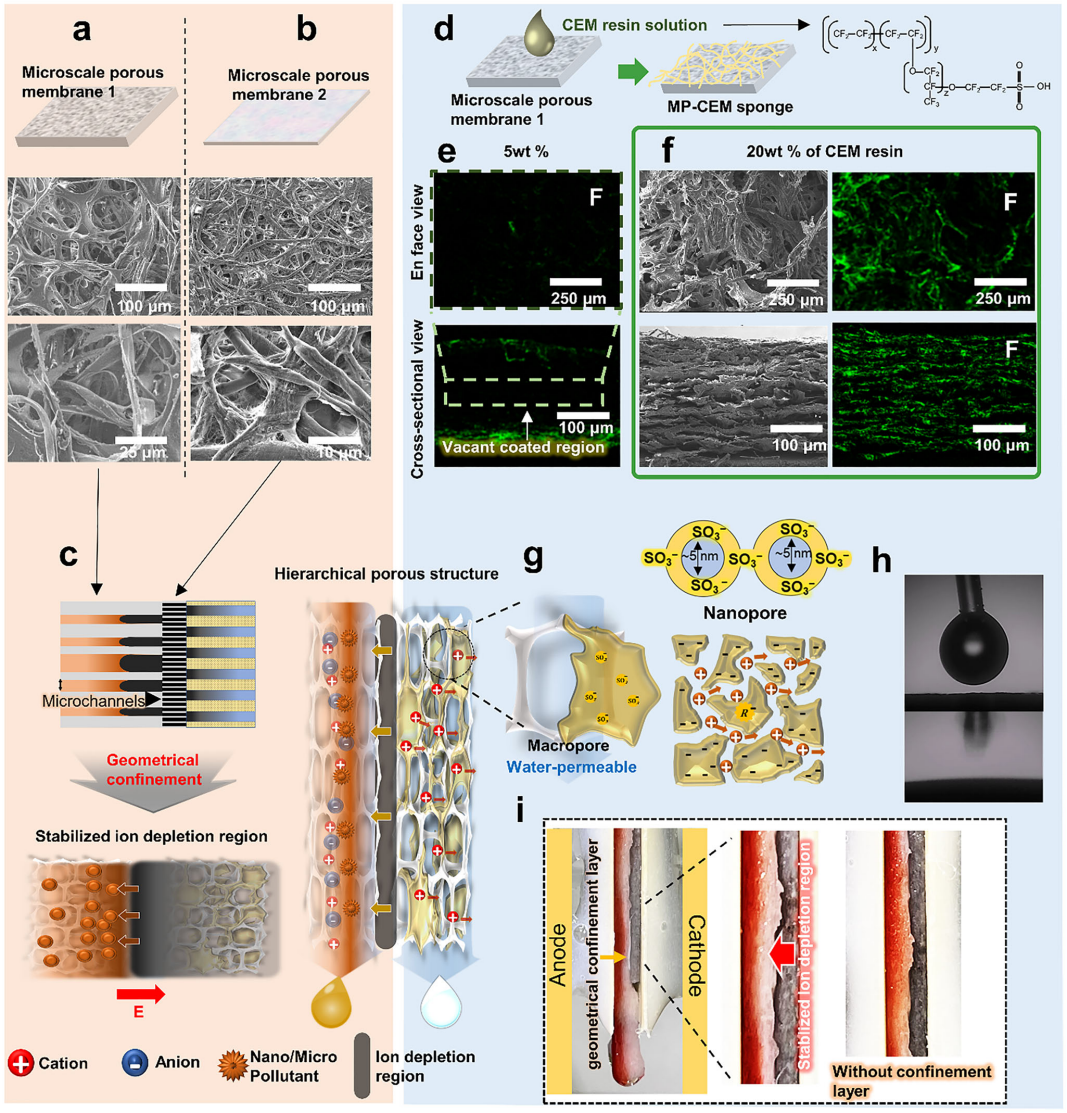
Design and structure of the hierarchical porous membrane used for scalable electrokinetic filtration. Scanning Electron Microscopy (SEM) images of the two microporous layers comprising the membrane structure. The top cellulose–cotton cloth (a) features broad macropores, while the intermediate filter paper (b) provides geometrical confinement with a narrower pore size. c) Schematic illustration of the stabilized ion‐depletion region formed by geometrical confinement in a microchannel‐like environment. d) Fabrication of the multiscale porous cation‐exchange membrane by coating of the sponge scaffold with a Nafion‐based cation‐exchange resin. e) Fluorine elemental mapping by EDS, showing poor a Nafion distribution with 5 wt% solution due to interfacial evaporation, which resulted in the generation of coating voids. f) SEM and EDS images showing the uniform surface and cross‐sectional Nafion coverage obtained using the 20 wt% solution, which formed an effective ion‐selective phase. g) Schematic representation of ion‐selective nanopores embedded within larger micropores, forming a multiscale ion transport network. h) Hydrophilic microstructured skeleton supporting a high water permeability (WCA  =  0°). i) Optical image of the stacked membrane within the electrokinetic module, showing a stabilized and an unstable ion depletion region due to incomplete assembly.

To overcome the scalability constraints typical of nanoelectrokinetic devices, the present study adopted a hierarchical porous‐membrane design, in which each layer fulfills a specific hydrodynamic and electrokinetic function. The first layer consists of pristine, hydrophilic cellulose‐cotton cloth, whose broad pore‐size distribution reaches ≈319 µm, with the bulk of the pore volume concentrated between 10 and 100 µm (**Figure** **2**a; Figure S2a, Supporting Information). Its intrinsic wettability ensures uniform fluid infiltration across the entire cross‐section, effectively eliminating the no‐slip‐induced parabolic Poiseuille profile that dominates conventional pipe flow. Under the design flux of 1000 L m^−2^ h^−1^ (assumed) and porosity with ε = 0.82, the permeability Reynolds number was determined to be Re√K ≈ 7 × 10^−^^3^, indicating that viscous drag dominates and inertial or shear‐diffusion corrections are negligible. A simple Darcy model, therefore, provides a valid estimate of the pressure drop, and the system can be regarded as exhibiting a relatively uniform velocity profile characteristic of plug flow even in the wide channels (Figure S3; Table S1, Supporting Information).^[^ ^42^, ^43^ ^]^

The second layer is a commercial cellulose filter paper with a comparatively narrow pore‐size range of 4–10 µm (Figure 2b). Although some local variation exists, this commercial filter paper is substantially more uniform than the first layer. It removes particulates larger than 10 µm and imposes sufficient geometric confinement to attenuate the spatial pore‐size heterogeneity of the first layer, thereby suppressing microscale vortex formation (Figure 2c). This confinement lowers the local Reynolds number and shifts the flow regime from electroosmotic instability (EOI) toward electroosmotic flow (EOF), consistent with Dydek's scaling theory.^[^ ^39^, ^44^ ^]^ By stabilizing the ion depletion zone, the layer supports sustained nanoelectrokinetic operations at practical macroscopic flow rates. The underlying mechanism of ion concentration polarization (ICP) and limitation of conventional H‐shaped electrokinetic system are illustrated in Figure S4 (Supporting Information).

The third layer was fabricated by coating the pristine sponge with a Nafion‐based cation‐exchange resin, which enables selective cation transport as the fluid flows through the microscale porous scaffold (Figure 2d). The presence of fluorine, indicative of Nafion incorporation, was confirmed using energy‐dispersive X‐ray spectroscopy (EDS), thereby verifying the formation of an ion selective exchange phase. The coating uniformity was influenced strongly by the solution viscosity, where in lower‐concentration formulations led to uneven deposition owing to rapid interfacial evaporation (Figure 2e),^[^ ^45^, ^46^ ^]^ whereas a 20 wt% Nafion solution produced a relatively uniform coating both internally and externally across the scaffold (Figure 2f; Figure S5, Supporting Information). This minimized surface accumulation and enabled consistent cation‐selective transport throughout the nanochannel of the active region (Figure 2g). Notably, the resulting membrane exhibited a multimodal pore‐size distribution spanning nanometer to micrometer scales. A distinct mesopore peak was observed between 5 and 15 nm, with a dV/dD value of ≈ 2.5 × 10^−3^ cm^3^ g^−1^ nm^−1^, reflecting the nanoconfined ion‐selective channels formed by the Nafion coating. Beyond the mesopore regime, the macropores detected in the micrometer range were predominantly distributed over the 10–100 µm range, with a porosity of 0.68 (Figure S2b; Table S1, Supporting Information), thereby preserving the inherent water permeability of the hydrophilic sponge (water contact angle, WCA = 0°), as shown in Figure 2h. Additional characteristics, including the thickness in both the dry and wet states, were measured, and the swelling ratio was calculated as the ratio of the wet‐to‐dry thickness (Table S2, Supporting Information).

This hierarchical pore architecture couples ion‐permselective nanopores with highly permeable macropores, thereby enabling complete wetting and field‐driven ion transport. Indeed, under an applied electric field, the hierarchical membranes established a stabilized ion depletion region near the MP‐CEM interface, driven by the differences in ionic mobility across the cation‐selective nanopores. This region exhibited an elevated local resistance, concentrating the electric field and generating a strong electrophoretic force that acted as an effective barrier against the downstream migration of negatively charged particles (Figure S4, Supporting Information). Notably, stable nanoelectrokinetic behavior was achieved over centimeter‐scale areas without the requirement for microchannel fabrication, as confirmed by anionic dye exclusion experiments (Figure 2i).

### 2.2 Nanoelectrokinetic‐Based Repulsion Performance using a Soluble Dye

**Figure 3:**
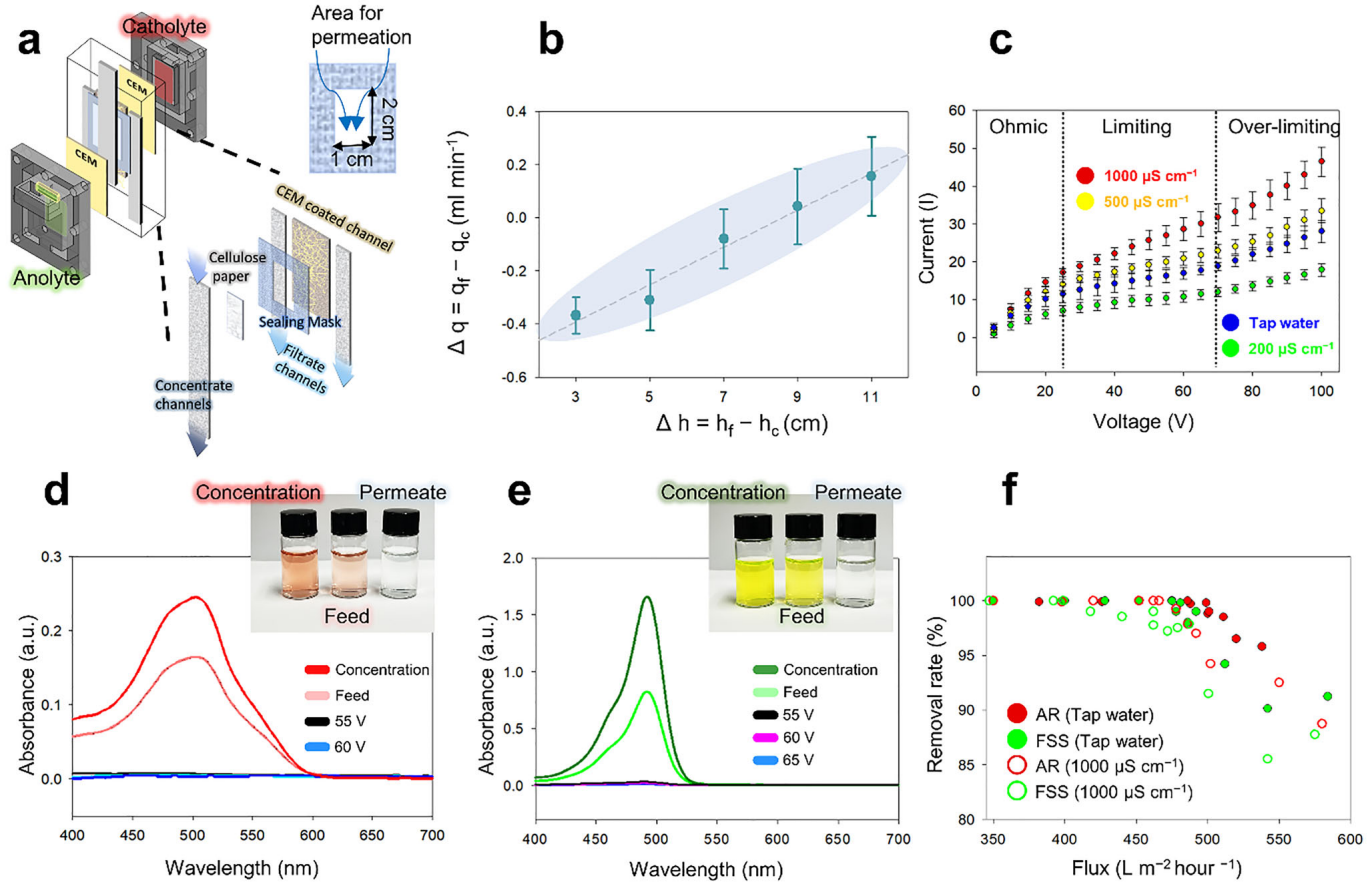
Nanoelectrokinetic performance of the centimeter‐scale filtration module under various flow and conductivity conditions. a) Schematic representation of the device architecture, showing the layered assembly with a system body, sealing the CEM for buffer compartmentalization. A hydrophobic flow mask is also incorporated, along with separate filtrate and concentration channels. b) Difference between filtrate and concentrate flow rates (Δq) as a function of outlet height difference (Δh), showing linear behavior and confirming Darcy‐based flow regulation under gravity‐fed conditions. c) Current–voltage (*I–V*) responses recorded from 0 to 100 V at different bulk conductivities (200–1000 µS cm^−1^), showing the canonical three regime behavior (ohmic, limiting, overlimiting) characteristic of ICP. d,e) UV–vis spectra and corresponding optical images of AR (50 ppm) and the FSS (10 ppm) under varying electric fields. f) Dye rejection performance as a function of the filtration flux under two conductivity conditions, confirming the trade‐off between the ionic strength and the flow‐driven breakthrough.

To evaluate the scalability of the nanoelectrokinetic phenomena, an electrokinetic filtration system was constructed comprising hierarchical main channels positioned between the anode and the cathode, wherein orthogonal concentration and filtration outlets were also incorporated (**Figure** **3**a). A 0.3 m Na_2_SO_4_ buffer was used as both the anolyte and the catholyte during application of the electric field. Since higher voltages may induce undesired electrochemical side reactions, the electrodes were placed in buffer reservoirs to confine these reactions and prevent their propagation into the main channel. To maintain ionic contact while preventing fluid leakage, the electrolyte reservoirs were isolated from the main channel using commercial cation‐exchange membranes. The effective filtration area was defined as 1 × 2 cm^2^ and confined using a patterned mask, as shown in Figure S6 (Supporting Information).

In multichannel systems composed of hierarchical porous media, conventional flow models are often difficult to apply owing to their structural heterogeneities, compression effects, and non‐uniform permeabilities. To address this issue, we adopted a passive flow control strategy based on gravitational head differences. Despite the variability across channels, each flow path individually obeys Darcy's law under the operating conditions. Consequently, the overall system behaves as a linear combination of independent hydraulic resistances, preserving global linearity and enabling predictable passive control of the flow distribution (Figure S7, Supporting Information)^[^ ^47^, ^48^ ^]^ Varying the height difference (inlet to outlet) between the concentrate (h_c_) and filtrate (h_f_) outlets (Δh = 3–11 cm) resulted in a proportional increase in filtrate flow rate (q_df_) (Figure 3b), indicating that an increased gravitational head governs the flow split according to the equation: Δ q = filtrate flow rate (q_f_) − concentration flow rate (q_c_). Experimentally, the flow‐rate difference between the filtrate and concentrate narrowed significantly at Δh values of 7–9 cm, suggesting a balance point in hydraulic resistance. Despite minor deviations attributed to porosity and compression, the obtained results demonstrate that linear flow regulation can be achieved reliably without external pumps by simply adjusting the channel height.

To verify the centimeter‐scale scalability of nanoelectrokinetic transport, current–voltage (I–V) curves were obtained between 0 and 100 V at bulk conductivities of 200–1000 µS cm^−1^. As expected for ICP systems,^[^ ^49^, ^50^, ^51^ ^]^ every curve showed the canonical three‐regime signature: i) An ohmic region (0–25 V) following Ohm's law, ii) a limiting region (25–70 V) in which the slope decreases as ion depletion leads to an increased interfacial resistance, and iii) an over‐limiting region (70–100 V) at higher voltages (Figure 3c). In the low‐conductivity buffer (200 µS cm^−1^) the limiting‐region slope decreased sharply, indicating the formation of a well‐defined iondepletion zone. At a higher conductivity (1000 µS cm^−1^), this slope reduction was less pronounced, as the shortened Debye length (<10 nm) inside the nanopores weakened cation selectivity and thus suppressed depletion‐zone development.^[^ ^50^, ^52^ ^]^ Unlike the plateau that is commonly observed in microfluidic chips, the charged porous scaffold continued to sustain an increasing current within the limiting regime, a behavior that is attributed to the surface conduction pathways in the matrix (Figure S8, Supporting Information).^[^ ^53^ ^]^ The transition to the overlimiting regime became more pronounced at higher conductivities, reflecting the activation of electroconvective or surface‐driven transport. Importantly, the reproducibility of this three‐regime profile across all tested conductivities confirmed that nanoscale electrokinetic phenomena can be stably sustained over centimeter‐scale areas under low‐salinity conditions representative of fresh water.

To validate the stability of ICP over centimeter‐scale porous systems, the electrophoretic exclusion of anionic dye molecules was evaluated under various electric field conditions. As shown in Figure 3d,e, two model dyes, namely Amaranth Red (AR, 50 ppm) and fluorescein sodium salt (FSS, 10 ppm) were tested under constant flow condition of 1.65 ± 0.12 mL min^−1^ (Δh = 7–9 cm). UV–vis spectral analysis revealed that AR, which exhibited a peak at 502.5 nm, was fully excluded at 55 V, with potentials of both 55 and 60 V achieving >99% dye removal. In contrast, the FSS, which peaked at 495.5 nm and carries fewer anionic functional groups than AR, required a higher threshold of 60 V to achieve a comparable exclusion. This difference is consistent with the lower quantity of negatively charged groups in the FSS structure compared to those of AR (Figure S9, Supporting Information). These results indicate that a well‐defined ion depletion region can be stably established across large areas, enabling the effective nanoelectrokinetic repulsion of dye molecules with nanometer‐scale dimensions.

To assess the effect of the throughput rate on the separation performance, the flow rate was varied, and the corresponding dye removal rates were measured (Figure 3f). In tap water solution conditions (320–380 µS cm^−1^), both >99% removal efficiencies were maintained for AR and FSS maintained >99% removal at filtration fluxes below ≈487 and ≈401 L m^−2^ h^−1^, respectively. Under elevated ionic strength (1000 µS cm^−^^1^) prepared by dissolving NaCl, the effective threshold fluxes decreased slightly to 462 and 388  L m^−2^ h^−1^, respectively, this was attributed to electrical double layer compression within the cation‐selective nanochannels. As the filtration flow exceeds the electrophoretic repulsion velocity, the dyes began to leak into the permeate stream, leading to reduced rejection. These results underscore the critical importance of flow regulation in maintaining the ICP performance and highlight the need for optimized operating conditions in large‐area systems. Notably, low‐conductivity solutions promoted stronger ion depletion and sustained the formation of effective electrokinetic barriers, confirming that ionic strength is a key determinant of nanoelectrokinetic behavior. Overall, this system demonstrates that nanoelectrokinetic filtration can be stably implemented over centimeter‐scale membranes, offering the promising potential for scalable water purification applications in low‐salinity solutions, such as those found in freshwater environments (e.g., rivers, streams, lakes, rainwater, and ponds).

### 2.3 Removal of Nanoscale SS for Water Purification

**Figure 4:**
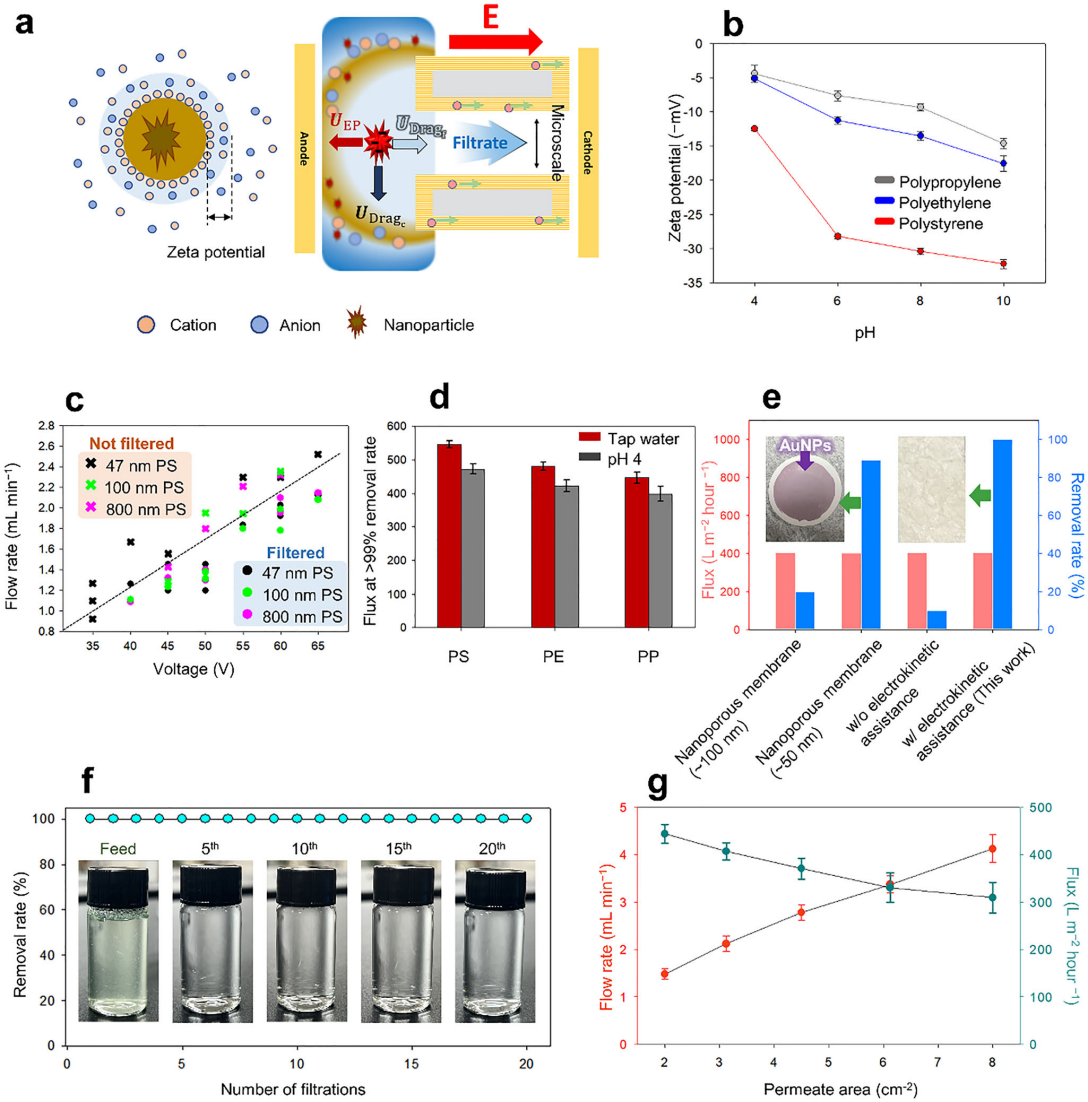
Nanoelectrokinetic removal of nanoplastics under various operating conditions. a) Schematic illustration of the electrophoretic exclusion mechanism under an applied electric field, showing the force balance among the electrophoretic velocity (U_EP_), the drag flow velocity of filtrate (U_Dragf_), and the concentrate (U_DragC_). b) Zeta potential (ζ) profiles of PS, PE, and PP microplastics measured over a pH range of 4–10. c) Removal performances for 47, 100, and 800 nm PS nanospheres under varying electric field strengths. d) Critical flux values at which PS, PE, and PP were removed at >99.9% efficiencies under neutral and acidic conditions. e) Comparison of nanoelectrokinetic filtration with commercial nanofiltration membranes (pore sizes: 100 nm and 50 nm) using 5–20 nm AuNPs. f) Reusability test over 20 filtration cycles using a 300 ppm microplastic mixture. g) Scalability assessment following active membrane area expansion (2–8 cm^2^).

To evaluate the ability of the system to remove nanoscale SS, three microplastics commonly found in natural water were employed, namely polystyrene (PS), polyethylene (PE), and polypropylene (PP).^[^ ^39^ ^]^ All three polymers exhibit negative zeta potentials in aqueous media, making them susceptible to electrophoretic exclusion under an applied electric field (*E*), wherein drag and electrophoretic forces dominate particle transport (**Figure** **4**a). According to the Smoluchowski relation,^[^ ^44^ ^]^ the steady‐state electrophoretic velocity (***U**_EP_*) of a particle with zeta potential (ζ) in a viscous medium is given as:

(1) $$U_{EP}=\frac{\varepsilon_{0}\varepsilon_{r}\zeta E}{\eta }$$

where 𝜂 is the dynamic viscosity of the fluid, ε₀ is the permittivity of free space, ε_r_ is the relative permittivity. Since ***U*** *_EP_* is proportional to both the local E and ζ, particles in the ion depletion region (where the electric field is amplified) experience maximum repulsion velocity, and the applied voltage determines the magnitude of this effect. For these anionic nanoplastics, zeta‐potential (ζ) measurements over pH 4–10 confirmed increasingly negative ζ‐values with rising pH, consistent with progressive deprotonation of surface groups (Figure 4b): PS registered the most electronegative range (−12.50 ± 0.25 to −32.26 ± 0.67 mV), PP fragments followed (−4.42 ± 1.25 to −14.63 ± 0.77 mV), while PE gave intermediate values (−5.12 ± 0.17 to −17.57 ± 1.16 mV). Given that typical freshwater and drinking water sources fall within pH 6.5–8.5 (WHO guideline), these results indicate that the device should maintain strong electrophoretic repulsion and achieve effective microplastic removal under environmentally relevant conditions.

Although the electrophoretic mobility can vary with particle size even among chemically identical microplastics, the proposed system was not designed to separate particles based on their differential mobilities within the confined channels. Instead, it operates by repelling suspended particles outward via an electrophoretic force under an applied electric field. In addition to the electrophoretic component, the particles also experience a drag velocity (***U***_Drag_) from the surrounding fluid flow. At low particle Reynolds number (Re ≪ 1), the particles follow Stokes'law under drag force induced by fluid flow. This ***U***_Drag_ is given as a function of time (t):

(2) $$U_{\mathrm{Drag}}\left(t\right)=\frac{F_{\mathrm{Drag}}}{6\pi \eta a}\;\left[1\;-\;\exp\left(\frac{-9\eta }{2\rho_{\mathrm{sph}}\cdot a^{2}}t\right)\right]$$

where η is the dynamic viscosity of the fluid, a is the particle's Stokes radius, and ρ_sph_ is the density of the spherical body. Since the particles are at the nanoscale, the relaxation time (τ_p_) is on the order of nano‐ to microseconds, as described in Figure S10 (Supporting Information). Thus, under experimental conditions, particle velocity can be approximated as fluid velocity (***U**_fluid_*),

(3) $$U_{Drag}\approx \;U_{fluid}=\frac{q}{A}$$

where q is flow rate and A is the cross‐sectional area of the effective filtration channel. And this ***U***_Drag_ can be divided into two components: ***U***_Dragc_, the fluid flow toward the concentration output, and ***U***_Dragf_, the permeation flow through the multiscale porous ion‐exchange membrane. Since only ***U***_Dragf_ acts opposite to the electrophoretic motion, we considered this term in the force‐velocity balance.

The balance between ***U***_EP_ and ***U***_Dragf_ was then used to interpret the observed particle rejection. To verify the robustness of this mechanism across a broad size range, 47, 100, and 800 nm PS nanospheres were tested. At 40 V, the system achieved >99% removal of these particles at a throughput of 1.26 mL min^−^^1^, and at voltages above 60 V, high removal rates were maintained even at elevated flows up to 1.92 mL min^−1^. As shown in Figure 4c, the dashed line represents the force balance the between 𝑈_Dragf_ and the 𝑈_EP_; effective filtration occurs below this line, where electrophoretic force dominates. All tested particle sizes followed similar filtration trends, indicating that despite their nanoscale dimensions, the particles were not separated based on size but were effectively excluded by electrophoretic force under the applied electric field.

In electrophoretic filtration systems, the extent of particle repulsion is proportional to the magnitude of the zeta potential of the particle. To examine this relationship, we evaluated the removal performance of PE and PP, since these polymers exhibit lower zeta potentials than PS under typical aqueous conditions. The corresponding experiments were performed at a total flow rate of 3.3 mL min^−^^1^ with a filtrate‐to‐concentrate ratio of ≈0.5 (± 10%) and an applied voltage of 60 V. As shown in Figure 4d, all three microplastics were effectively excluded at fluxes near 400 L m^−2^ h^−1^ under both neutral (tap water) and acidic (pH 4) conditions. Consequently, >99.9% removal of PS was achieved at fluxes of 472 L m^−2^ h^−1^ (pH 4) and 532 L m^−2^ h^−1^ (tap water), and the complete rejection of PE and PP was also achieved at slightly lower flux thresholds of 422–482 and 398–446 L m^−2^ h^−1^, respectively. The findings confirm that electrophoretic repulsion enables the efficient removal of nanoplastics across a range of surface charges. Notably, even sub‐10‐nm particles (Figure S11, Supporting Information) were completely excluded using a membrane composed of micrometer‐scale pores, without the requirement for nanoscale sieving structures.

Subsequently, we benchmarked the proposed system against a conventional size‐based nanofiltration using gold nanoparticles (AuNPs, 5–20 nm), which exhibited distinct UV–vis absorption peaks (*λ*_max_ = 525 nm) to enable accurate quantification (Figure S12, Supporting Information). At a flux of ≈400 L m^−2^ h^−1^ under a gravity‐driven flow (<1 kPa) and an applied electric field, the current system successfully excluded the NPs without relying on nanoscale pores. In contrast, the commercial nanofiltration membranes with nominal pore sizes of 100 and 50 nm failed to achieve complete removal under similar operating conditions (404 and 402 L m^−2^ h^−1^, respectively) (Figure 4e). A comparative summary between the electrokinetic filtration system and previously reported nano‐ and ultrafiltration technologies is presented in Table S3 (Supporting Information). Importantly, while most commercial nano‐ and ultrafiltration systems operate at relatively low flux levels (≈150 L m^−2^ h^−1^) and require high‐pressure pumps, our system achieves ultrafine particle removal at much higher throughput (≈400 L m^−2^ h^−1^).

Unlike pressure‐driven systems that rely on strict pore‐size exclusion, the nanoelectrokinetic mechanism of the current design employs electrophoretic repulsion to reject the NPs, thereby significantly reducing the risk of pore‐clogging and fouling, as illustrated in the inset images in Figure 4e and Figure S13 (Supporting Information). The findings demonstrate that effective NP removal can be achieved using simple micrometer‐scale porous structures without the need for high‐pressure pumps. Moreover, unlike size‐based sieving, the electrophoretic mechanism prevents pore clogging, thereby enabling membrane reuse without causing irreversible fouling (Figure S14; Table S4, Supporting Information). In real surface water, scaling factors such as NOM, silicates, and hardness (Ca^2^⁺, Mg^2^⁺) must be considered. Humic acid is particularly challenging due to its polydisperse, aggregating nature and non‐monotonic charge–size relationship.^[^ ^54^ ^]^ Tests conducted for 3 h per day over three consecutive days showed that humic acid removal gradually declined from nearly 100% to 96.12 ±1.21%, while the flux was maintained ≈ 200 L m^−^^2^ h^−1^ (Figure S15a, Supporting Information). In contrast, silica, calcium, and magnesium were transported through CEM under applied electric field without significant accumulation (Figure S15b; Table S5, Supporting Information). Nevertheless, as a negatively charged natural organic matter (NOM) fraction, humic acid was not completely removed but was effectively repelled, suggesting a distinct antifouling advantage over conventional membrane filtration.

The reusability of the hierarchically structured MP‐CEM is essential for practical applications. Thus, to evaluate its long‐term performance, 20 consecutive filtration cycles were performed using a 300 ppm mixed nanoplastic suspension (PS, PP, and PE; 100 ppm each), with the system disassembled and all channels thoroughly cleaned between cycles, and 10 mL of permeate collected per cycle under identical operating conditions. As shown in Figure 4f, removal efficiencies remained consistently above 99.9%, as confirmed by UV–vis absorbance (Figure S16, Supporting Information). Moreover, the incorporation of a microscale porous, hydrophilic cellulose scaffold enabled facile surface restoration on rinsing with water, demonstrating the washability and reusability of the membrane. This antifouling characteristic supports stable and repeatable operation over multiple cycles.

To evaluate the scalability of the nanoelectrokinetic filtration platform, the effective membrane area was increased while maintaining a fixed 1:2 (width:height) aspect ratio for the filtrate‐facing region. As shown in Figure 4g, expanding the effective membrane area from 2 to 4 cm^2^ resulted in a proportional increase in total flow rate from 1.48 ± 0.11 to 4.20 ± 0.29 mL min^−1^. Although the flux decreased moderately from 444.02 ± 32.62 to 309.38 ± 22.08 L m^−2^ h^−1^ (i.e., a typical trend observed in scaled‐up fluidic systems) the removal efficiency remained consistently > 99.9% under all conditions. In addition, the expanded system also exhibited a stable ion depletion region for more than 7 h, as verified by the current–time responses shown in Figure S17 (Supporting Information). The initial sharp change in current is attributed to the propagation and subsequent convergence of the ion depletion region, leading to a quasi‐steady state. Once this state is reached, the current gradually decreases over time as a consequence of continuous ion depletion without buffer replenishment. Notably, compared to systems lacking a stable confinement layer where the ion depletion region remains unstable, our system with fully developed layers exhibits relatively minor fluctuations, thereby demonstrating more stable current behavior. These results confirm that the applied electric field sustains uniform electrophoretic exclusion across the expanded membrane area, allowing effective scale‐up without compromising performance. A general comparison of other electrokinetic and electrochemical methods for fine particle separation is shown in Table S6 (Supporting Information). In this respect, identifying the optimal effective surface area that maximizes the permissible inlet flow rate and expanding the system in parallel under such conditions would enable a substantial increase in total throughput. Moreover, the cross‐flow configuration not only minimizes cake layer formation and reduces clogging^[^ ^55^, ^56^ ^]^ but also promotes a uniform electric field distribution in conjunction with the vertically aligned field geometry, thereby highlighting the feasibility of this system for use in large area nanoelectrokinetic water treatment applications.

### 2.4 Solar‐Powered Nanoelectrokinetic Filtration Test

**Figure 5:**
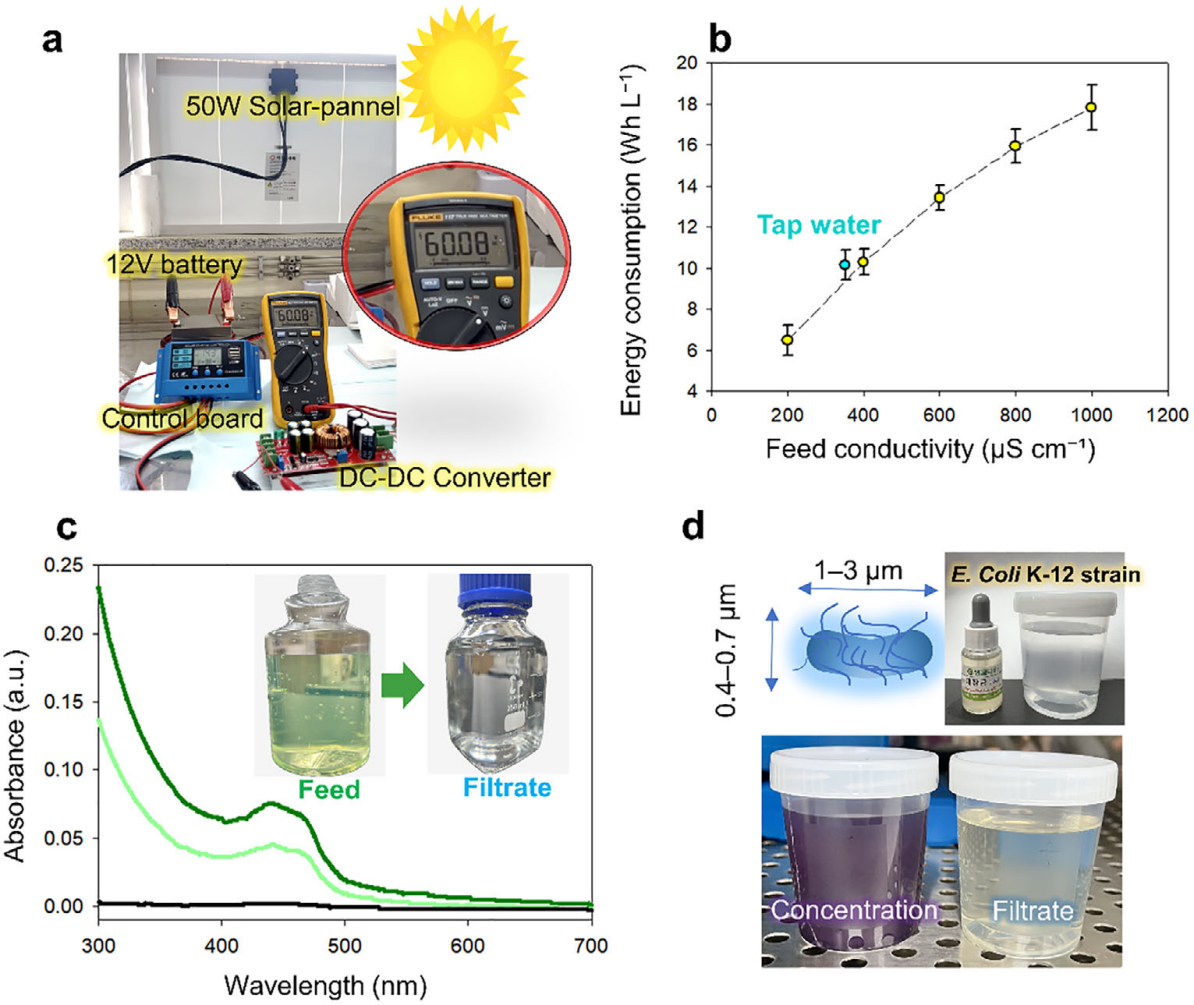
Solar‐powered operation and practical performance validation of the nanoelectrokinetic filtration system. a) Setup of the compact solar‐driven power module. b) SEC as a function of the feed conductivity (using tap water and NaCl, 200–1000 µS cm^−1^). c) UV–vis spectra of the feed and filtrate after 1 h solar‐powered operation with PS nanoplastics. d) Visual detection of *E. coli* using the enzymatic colorimetric assay. The permeate remained clear, indicating bacterial levels below the detection limit (<1 CFU/100 mL).

Unlike NF/RO units that depend on power‐intensive pumps, our system operates without such components. For example, commercial portable RO devices typically demand 36–400 W of output power (see Table S7, Supporting Information for comparison), whereas our electrokinetic platform functions effectively at <10 W. To meet the demand for solar‐powered water purification, a compact power module was assembled in which a 50 W photovoltaic panel charged a 12 V buffer battery via a charge controller, which also allowed monitoring of the input voltage and battery status. The battery was incorporated to stabilize the power supply and ensured a consistent voltage delivery under fluctuating sunlight conditions. A direct current (DC)–DC boost converter was subsequently used to provide a stable 60 V input to the nanoelectrokinetic unit (**Figure** **5**a). The lab‐scale prototype featured a 2 × 4 cm^2^ permeate membrane. The feed solution was delivered using a Mariotte bottle, which passively regulated the hydrostatic pressure and maintained a steady volumetric flow (Figure S18, Supporting Information). A vertical height difference of 10 cm between the inlet and outlet, resulted in a flow rate of ≈8 mL min^−^.^1^

Since the nanoelectrokinetic stack is the only electrical load in the solar‐powered setup, and no pumps or auxiliary devices were incorporated to draw power, the measured specific energy consumption (SEC) effectively represents the total energy cost of the process. Thus, the SEC was quantified by filtering gravity‐fed NaCl solutions spanning the potable‐water conductivity range (200, 500, and 1 000 µS cm^−^^1^) under a constant applied voltage of 60 V. As shown in Figure 5b, SEC increased quasi‐linearly from 6.48 ± 0.74 Wh L^−^^1^ at 200 µS cm^−^^1^ to 17.8 ± 1.11 Wh L^−^^1^ at 1000 µS cm^−^,^1^ consistent with the Ohmic decrease in membrane resistance, while removal efficiency remained > 99.9% across all conditions. More specifically, tap‐water conditions (≈ 350 µS cm^−^^1^), the SEC was 10.14 ± 0.72 Wh L^−^,^1^ corresponding to a steady‐state power draw of just ≈2.4 W due to the low operating current, despite the high applied voltage. Considering the full system efficiency (photovoltaic generation: 65%, battery charging: 95%, DC–DC voltage conversion: 90%), a 50 W solar panel operating for 4 h under average sunlight yielded ≈110 Wh of usable energy, as described in Table S8 (Supporting Information). Theoretically, this is sufficient to purify ≈11 L of tap water. While the actual performance may vary according to the environmental conditions, the obtained results demonstrate that the energy demand scales predictably with the ionic strength and remains well within the capabilities of a compact off‐grid solar infrastructure.

To assess the ability of the system to sustain a long‐term removal performance under solar‐powered operation, a 1 h continuous filtration test was conducted using PS nanoplastics, as the model contaminant. The UV–vis spectra of the permeate were recorded after 1 h, and the buffer reservoir was replenished to its initial level after each sampling to maintain consistent hydrostatic conditions across repeated runs. Throughout the experiment, the solar‐powered circuit maintained 60 V (±0.2 V) consistently, and the filtrate absorbance remained at baseline levels, indicating > 99.9% particle rejection (Figure 5c). These results confirm the capacity of the system to provide stable removal performance in off‐grid settings. Additional experiments using mixed suspensions containing PP and PE, as well as removal of organic dye (AR) under identical conditions, further support the generalizability of this platform (Figure S19, Supporting Information).

Pathogenic bacteria, such as *E. coli* pose a major barrier to the use of untreated freshwater, leading to waterborne diseases. Additionally, these bacteria are major contributors to membrane biofouling. Similar to the majority of microbial contaminants in natural waters, *E. coli* exhibits a negative zeta potential,^[^ ^57^, ^58^ ^]^ rendering it susceptible to electrophoretic exclusion under an applied electric field. As detailed in Figure S20 (Supporting Information), a chip‐based system confirmed that *E. coli* can be effectively repelled by ICP mechanisms. To evaluate both the disinfection capability and antifouling potential of the nanoelectrokinetic module, we conducted a 1 h filtration test using an *E. coli* suspension (*E. coli* K‐12) under a gravity‐driven flow and a constant 60 V bias. Samples were collected every 30 min and analyzed using the enzyme–substrate method, which allowed the colorimetric detection of both *E. coli* and the total coliforms (i.e., the enzyme substrate coliform method) in 100‐mL samples via colorimetric observations after 24 h incubation at 35 °C. Specifically, the presence of β‐galactosidase–positive coliforms hydrolyzed the chromogenic substrate M‐Gal (6‐chloro‐3‐indolyl‐β‐D‐galactopyranoside) to produce a magenta pigment, indicating contamination, whereas the absence of such activity resulted in a clear solution. Once the concentrate developed a violet hue, the permeate remained clear throughout the test (Figure 5d), thereby confirming that the bacterial levels were below the detection limit (<1 CFU/100 mL). These results demonstrate the capacity of the developed system for effective microbial removal, while also confirming its inherent resistance to biofouling, and reinforcing its potential for application in the treatment of bacterially contaminated freshwater.

## 3 Conclusion

This work demonstrates that ultrafine particles even within the sub‐10‐nm range can be effectively removed using a micrometer‐scale porous membrane without the necessity for nanoscale pores or pressure‐driven nanofiltration. Through the integration of hierarchical structures (i.e., hydrophilic cellulose cloth, size‐confining filter paper, and a Nafion‐coated ion‐exchange sponge), the nanoelectrokinetic phenomena was extended to centimeter‐scale, during a gravity‐fed operation. Notably, the developed system achieved >99.9% removal of micro/nanoplastics and led to undetectable E. coli levels (<1 CFU/100 mL) at 60 V and <1 kPa, while maintaining a high permeate flux of ≈400 L m^−2^ h^−1^ under gravity‐driven conditions. The specific energy consumption ranged from 6.48 to 18 Wh L^−1^, wherein this consumption depended on the feed conductivity (≈10 Wh L^−1^) for tap water. Additionally, it was demonstrated that a 50 W solar panel operating for 4 h was able to purify ≈11 L of water. Moreover, this system maintained a comparable performance upon scale‐up from the effective membrane area of 2 to 4 cm^2^, and endured 20 reuse cycles without fouling. Overall, these results confirm the fabrication of a scalable, solar‐compatible, and pump‐free filtration platform that exhibits strong potential for off‐grid water treatment.

For further study, a robust hydrophilic cation‐exchange membrane with a microscale porous mesh structure will be essential for enhancing the durability and operational stability of the filtration system. The development of fluorine‐free CEMs should also be pursued as a sustainable alternative, given that this remains a global challenge. In addition, future research will incorporate a multiscale porous anion‐exchange membrane to address cationic pollutants. Subsequent work will explore integrated treatment strategies to address natural organic matter, which remains inefficient under single tpye precess. In parallel, efforts will focus on optimizing current leakage, cell resistance, and parasitic energy losses to improve the overall energy efficiency of the system.

## 4 Experimental Section

### Materials and Reagents

All materials used in this study were commercially available. The biodegradable, plant‐derived cellulose‐sponge membrane (Swedish dish cloth, Sprout, USA) was used as the macroporous support. A size‐confining intermediate layer was added using Whatman Grade 1 filter paper (Cytiva, USA) with a nominal pore size of ≈10 µm. The ion‐selective layer was formed by coating a 20 wt% Nafion dispersion (D2020, DuPont, USA) onto the membrane surface. A commercial cation‐exchange membrane (CMHPES 56102, Ralex, MemBrain sro, Czech Republic) was incorporated at both the anode and cathode sides of the electrochemical cell. Fluorescent PS nanospheres with nominal diameters of 47, 100, and 800 nm were obtained from Thermo Scientific (USA), while PE microparticles were sourced from Cospheric LLC (USA), and the PP fragments were purchased from Polysciences Inc. (USA). The AuNPs (5–20 nm, citrate‐stabilized suspension) were purchased from Sigma‐Aldrich. The solution conductivity was adjusted using analytical‐grade sodium chloride (NaCl, Samchun Chemicals, Republic of Korea). Humic acid and sodium metasilicate pentahydrate were obtained from Sigma–Aldrich (USA). Calcium chloride (CaCl_2_) and magnesium sulfate pentahydrate (MgSO_4_·5H_2_O) were purchased from Samchun Chemicals (Republic of Korea). For bacterial testing, an *E. coli* K‐12 strain was obtained from Sciencetool (Republic of Korea), and detection was performed using the ColiDuo enzyme–substrate kit (Microgiene, Republic of Korea).

### Membrane Fabrication

The composite membrane was fabricated by sequentially stacking three layers to form a three‐layer composite membrane. More specifically, these layers consisted of a hydrophilic cellulose cloth (Sprout dish cloth), Whatman 1 filter paper (10 µm nominal pore size), and the Nafion‐coated ion‐exchange sponge. A 20 wt% Nafion dispersion (D2020) was applied to the sponge surface by pipetting 0.4 mL cm−2 of the solution evenly across the active area. To prevent wrinkling or deformation during curing, the four corners of the membrane were secured with clips while drying at 60 °C. The Nafion layer was extended ≈0.5 cm beyond the intended permeation window to ensure full edge coverage. The effective membrane area was defined using a non‐woven release liner with a medical‐grade Tegaderm film (3 M) affixed over it, preventing leakage or wicking into surrounding regions during long‐term operation.

### Physicochemical Characterization

The surface and cross‐sectional morphologies were imaged using field‐emission scanning electron microscopy (FE‐SEM, JSM‐7401F, JEOL, Japan). The elemental fluorine distribution in the Nafion layer was visualized by EDS on the same instrument. The static WCAs of the pristine filtration membrane and the composite porous filtration membrane were measured using a SmartDrop goniometer (FEMTOBIOMED Inc., Republic of Korea). The membrane thicknesses were determined using a digital micrometer (Mitutoyo, Japan), and the pore‐size distributions were obtained by mercury intrusion porosimetry (Autopore V 9620, Micromeritics, USA). The zeta potentials and hydrodynamic diameters of all particle suspensions were characterized using a Zetasizer Nano‐ZS instrument (Malvern PANalytical, UK), while the solution pH was monitored using a portable pH meter (HI1271, Hanna Instruments, Republic of Korea).

### Electrokinetic Filtrtation Module

The filtration cell body was fabricated using a 3D printer (Formlabs, Somerville, MA, USA). Separate anolyte and catholyte chambers were filled with 0.3 m Na_2_SO_4_ as the supporting electrolyte, and each chamber was hydraulically isolated from the central feed channel using a commercial CEM (CMHPES 561 02, Ralex). The membrane perimeter was sealed with 30‐min epoxy adhesive (CEMEDINE, Japan) to prevent cross‐leakage. The anode consisted of a titanium mesh (100 µm wire diameter, 70% open area), while the cathode was constructed from carbon paper electrically bonded to a titanium lead wire to minimize contact resistance. The flow rate was passively regulated using a height‐adjustable inlet reservoir and an overflow outlet to establish a gravity head (Δh ≈ 10 cm). A pristine hydrophilic cellulose cloth or melamine sponge sheets were placed at the outlet to regulate the flow rate. The total thickness of the hierarchical channels, defining the electrokinetically active region, was fixed at ≈3.5 mm to ensure a consistent electric field strength.

### Analytical Methods

UV–vis standard curves were established for aqueous dispersions of the anionic dyes, PS, PP, PE, AuNPs, and humic acid. The particle removal efficiency (E) was calculated using the equation E = (C_0_ − C_1_)/ C_0_, where C_0_ and C_1_ represent the concentration of the original feed dispersion and the filtrate, respectively, expressed in mg L^−1^. For the nanoplastic samples, quantitative determination of the concentration based on the peak absorbance was often unreliable due to signal limitations. In such cases, UV–vis spectroscopy was used to qualitatively assess for the presence or absence of particles. These results were also cross‐validated using dynamic light scattering to confirm the presence of residual nanoparticles in the permeate samples.

### Filtering Speed

Peristaltic pump was used to deliver the feed to the inlet, maintaining a constant flow rate without applying additional pressure. To determine the filtrate flow distribution, the weight of the collected filtrate was measured, assuming a density of 1 g mL^−1^.

### Reproducibility Test

All measurements were conducted at least in triplicate to ensure reproducibility. For all repeated experiments, including the 20‐cycle reusability test, the filtration system was disassembled and each channel was cleaned before every run. The cleaning procedure was straightforward, as the microscale porous channels allowed easy removal of residual particles. Specifically, the channels were rinsed with running tap water or soaked in water overnight, while the MP‐CEM was additionally post‐treated by flowing clean water under an applied electric field to electrokinetically repel remaining particles. This process ensured consistent cleaning and continuous operation without the need for membrane replacement.

## Conflict of Interest

The authors declare no conflict of interest.

## Author Contributions

W.C. conceived the original idea, developed the analytical methodology, and wrote the initial draft of the manuscript under the supervision of G.L. M.L. contributed to the refinement of the methodology and provided critical feedback on the experiments and data analysis. W.C. and Y.J.P. conducted the experiments and analyzed the data. S.Y. prepared the schematic illustrations. All authors discussed the results, contributed to the manuscript preparation, and approved the final version for submission.

## Supporting information



Supporting Information

Supplemental Video 1

Supplemental Video 2

## Data Availability

The data that support the findings of this study are available from the corresponding author upon reasonable request.
