# Supplementary material for: Solar‐Powered Electrokinetic Filtration using Hierarchical Porous Membranes for the Off‐Grid Removal of Ultrafine Contaminants
Source: Adv Sci (Weinh). 2025 Oct 14;13(6):e15435. doi: 10.1002/advs.202515435 (PMC12866875; doi:10.1002/advs.202515435)
Supplement: Supplementary file 1 — Supporting Information [file ADVS-13-e15435-s002.docx]

Supporting Information

**Solar-powered Electrokinetic Filtration Using Hierarchical Porous Membranes for Off-Grid Removal of Ultrafine Contaminants**

*Woonjae Choi^a^, Minsoo Lee^a^, Young June Park^a^, Seungbin Yoon^a^, and Geunbae Lim^*a^*

*a Department of Mechanical Engineering, Pohang University of Science and Technology (POSTECH), 77 Cheongam-Ro, Nam-Gu, Pohang, Gyeongbuk 37673, Republic of Korea*

* Corresponding author: Geunbae Lim

Email: [limmems@postech.ac.kr](mailto:limmems@postech.ac.kr)


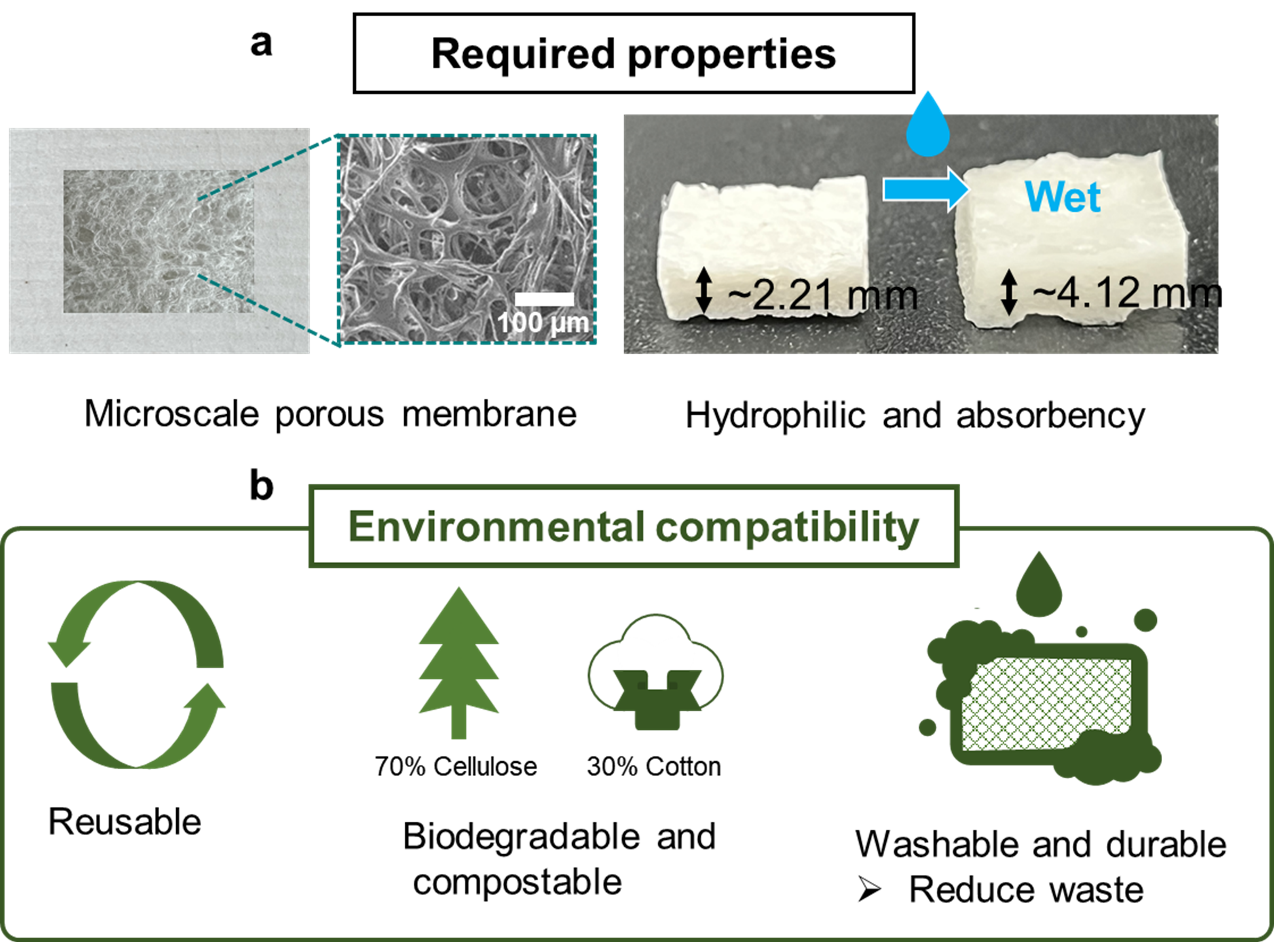


**Figure S1** Essential material properties and environmental compatibility for sponge-based applications. (a) Representative and SEM images show the porous, fibrous microstructure that enables fluid permeability and mechanical robustness. (b) Environmental compatibility is characterized by key attributes such as biodegradability and the ability to be reused after contamination and washing


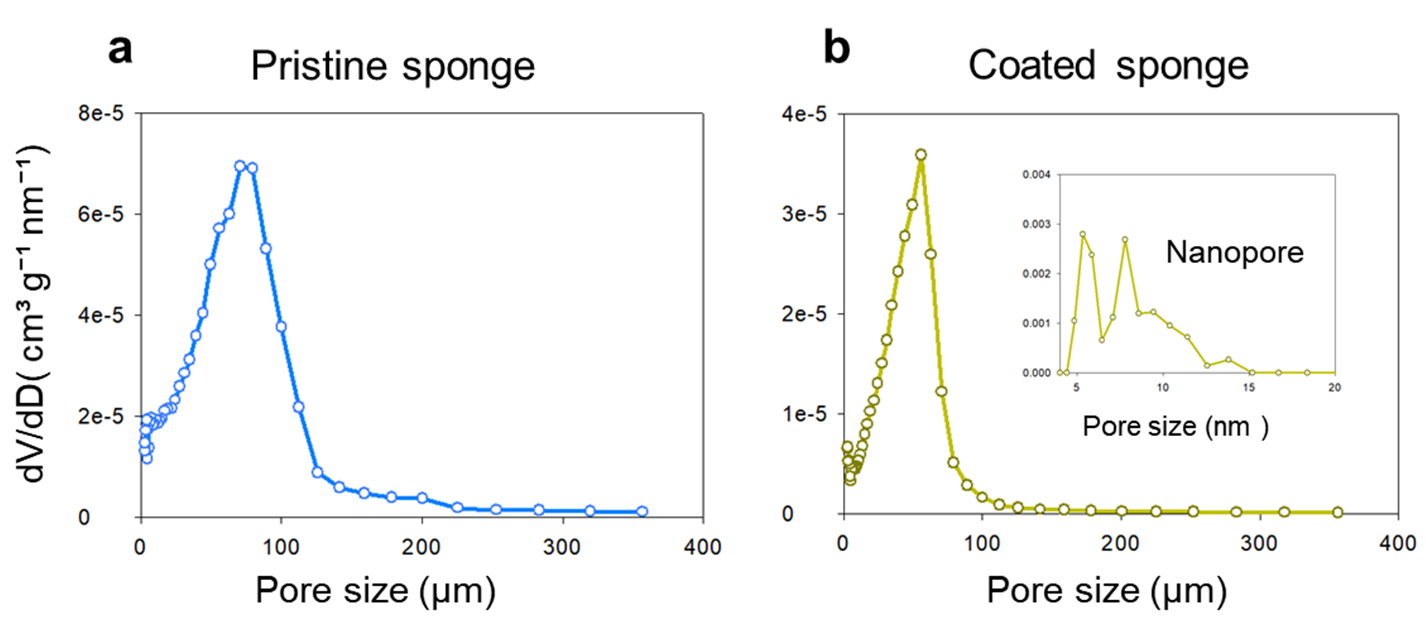


**Figure S2** Pore size distribution (dV/dD) profiles analyzed via mercury intrusion porosimetry. (a) The blue sample exhibits a dominant macroporous structure, with pore sizes extending up to ~319 μm and the majority of the pore volume concentrated in the 10–100 μm range.
(b) The yellow sample shows a primary microscale-pore peak (~60 µm) and additional nanopores attributed to the Nafion ionomer, which forms fixed cation-exchange domains. The inset reveals a broader nanopore distribution (4–15 nm), indicating a hierarchical structure.


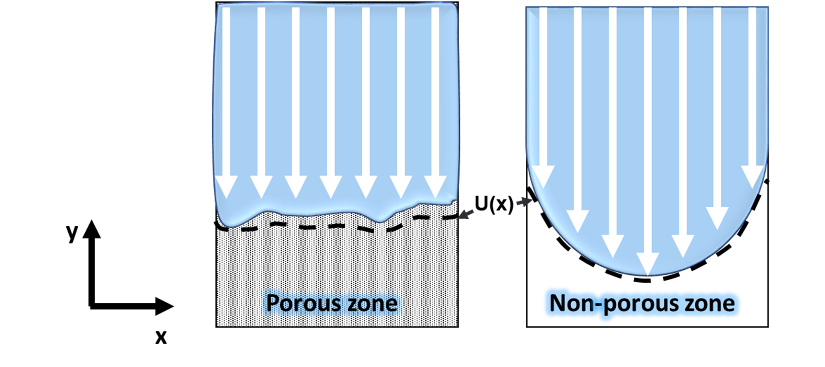


**Figure S3** Comparison of fluid infiltration and spreading behavior on porous and non-porous substrates. Schematic representation of Darcy flow through a porous medium. The velocity profile becomes nearly uniform (plug-like) due to the spatial averaging of pore-scale flow fields under low Reynolds number conditions. A fully developed parabolic velocity profile u(x) is observed in the non-porous channel section.

To evaluate the dominant flow regime in the sponge layers, we calculated the permeability Reynolds number (*Re_K_*), which characterizes the relative contribution of inertial and viscous effects in porous media flow

$${Re}_{K}= \frac{U\sqrt{K}}{\nu}$$

where *U* is the characteristic velocity, *K* is the permeability of the medium, and *ν* is the kinematic viscosity. *Re_K_* ≪1: Viscous forces dominate, and inertial effects are negligible. Under these conditions, the Darcy law is sufficient, describing a linear relationship between pressure gradient and flow velocity [1].

The permeability *K* of the sponges was estimated using the Kozeny–Carman relation:

$$K= \frac{\varepsilon^{3}d_{p}^{2}}{180{(1-\varepsilon)}^{2}}$$

where *𝜀* is the porosity, and *d* is the mean pore diameter, assumed to be 50~100 μm based on SEM imaging and mercury intrusion porosimeter.

**Table S1** Estimation of Permeability-Based Reynolds Number for Pristine and Coated Sponges

|  | Permeability Reynolds Number Estimation | | | |
| --- | --- | --- | --- | --- |
|  | Porosity (*𝜀*) | Permeability (K) | Velocity (U) | *Re_K_* |
| Pristine sponge | 0.82 | 6.32e-10 | 2.78e-4 m s^-1^  (1000 L m^-2^ h^-1^) | 0.007 |
| Coated sponge | 0.68 | 1.76e-10 |  | 0.0037 |

Since both values are well below 1, the flow in these porous scaffolds is dominated by viscous resistance, and Darcy’s law provides a valid and sufficient description of the pressure–velocity relationship.


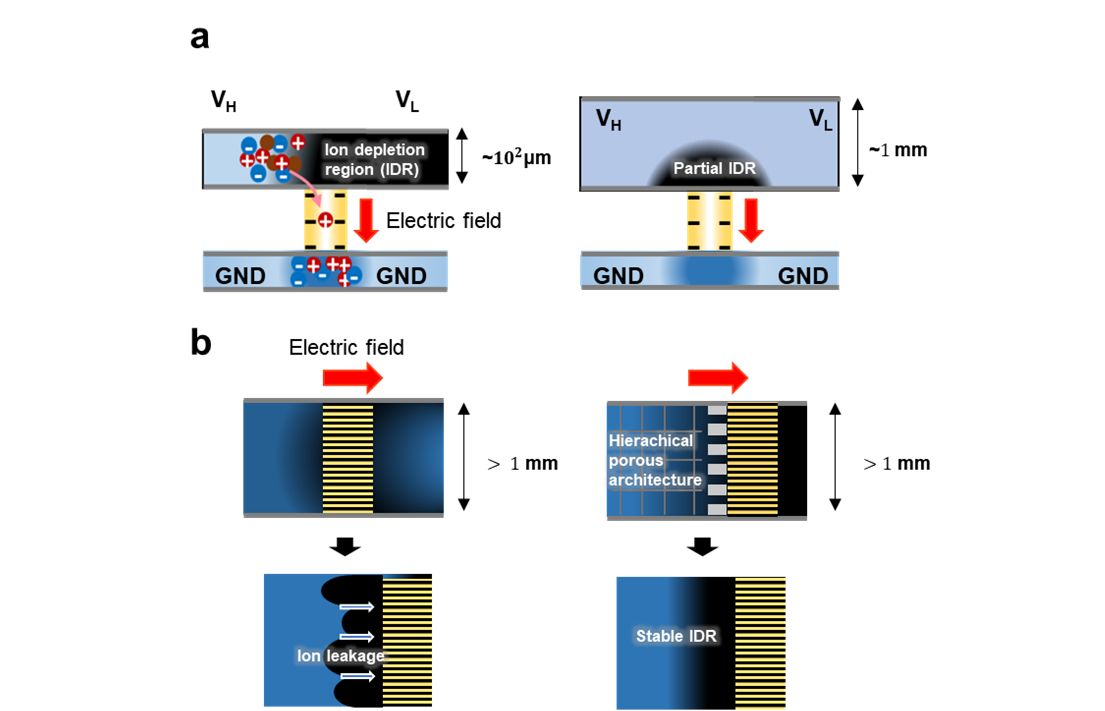


**Figure S4** Schematics illustrating ion depletion region (IDR) formation in (a) a typical AEM-bridged H-shaped electrokinetic system exhibiting partial and unstable IDR due to limited field confinement, and (b) the proposed system incorporating a hierarchical porous architecture to support full-scale and stable IDR formation for effective electrokinetic operation.

Ion concentration polarization (ICP) is an electrokinetic effect that arises from the ion-selective transport behavior of charged nanoporous membranes. When a direct current (DC) electric field is applied, counter-ions migrate through the membrane, while co-ions are excluded. This selective transport causes a concentration imbalance near the membrane interface, forming an ion depletion region (IDR) characterized by a steep drop in ionic strength and a locally enhanced electric field. The resulting field acts as a repulsive electrokinetic barrier to co-ions and negatively charged species, effectively inhibiting their passage.

In a conventional H-shaped hybrid channel structure, a uniform ion depletion region can be induced across the channel width up to several hundred micrometers. However, in millimeter-scale wide channels, the depletion zone fails to extend across the entire channel. As a result, particles can escape through regions not reached by the depletion layer, where the locally amplified electric field is absent and electrokinetic repulsion becomes ineffective. Moreover, as the channel cross-section increases, the system becomes susceptible to electrokinetic instabilities, most notably the emergence of three-dimensional helical vortices ^[2]^. These disturbances disrupt the stability of the ion depletion region and diminish particle separation efficiency.

To address this limitation, we designed a hierarchical porous channel with MP-CEM integrating nanopores and micropores. The MP-CEM support ion transport as in conventional CEM, while the micropores permit transverse fluid flow. With the hierarchical channels providing geometrical confinement, parallel microchannels are effectively created. This architecture allows localized depletion region to merge into a uniform layer across the channel width, thereby enhancing and homogenizing the electric field for effective particle manipulation in wide channels.


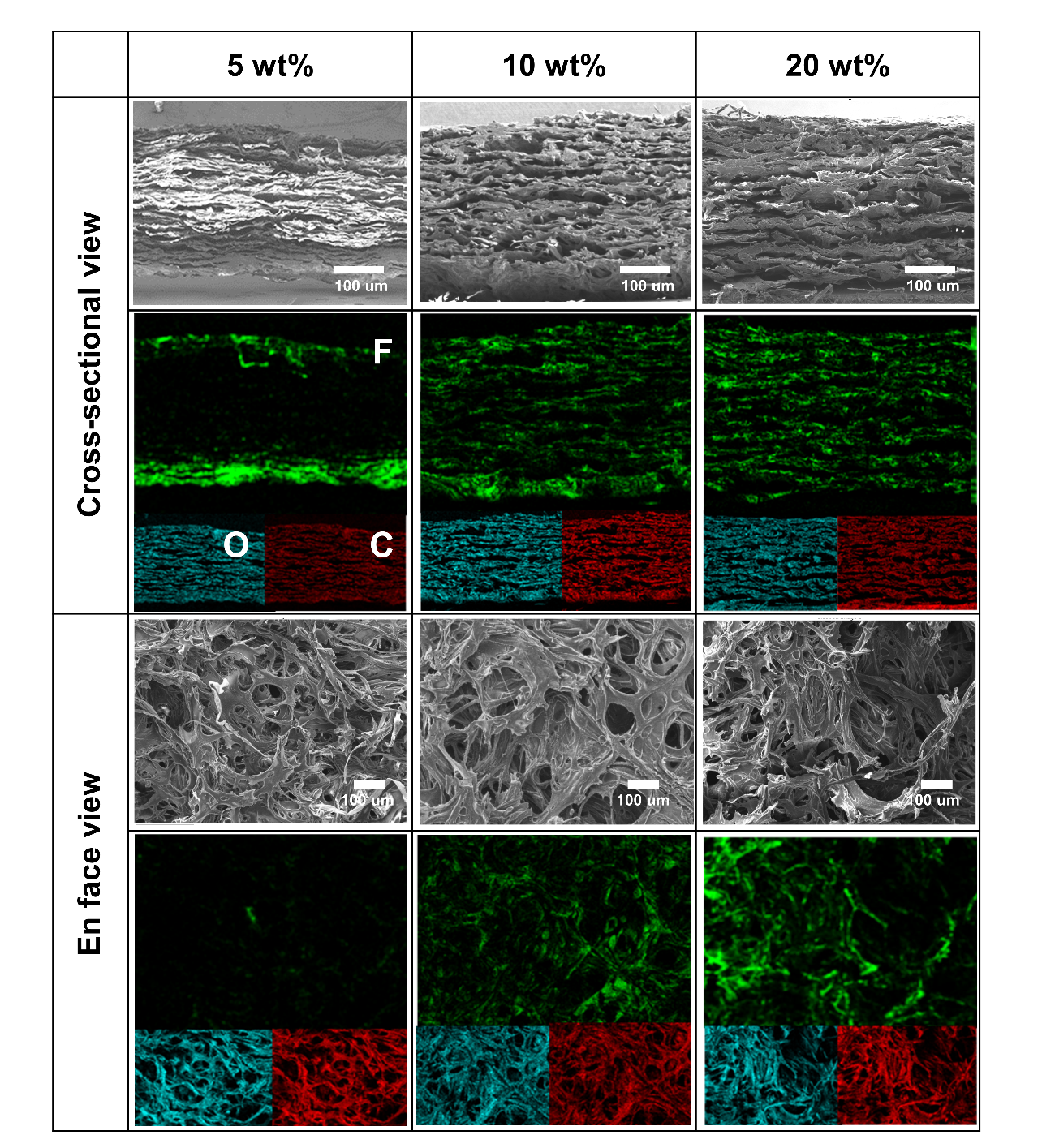


**Figure S5** SEM and EDS analyses of Nafion-coated ion-exchange multiscale porous composite sponges under varying coating conditions. Cross-sectional and en face EDX mapping images of the middle layer show the elemental distribution of fluorine (green), oxygen (cyan), and carbon (red).

**Table S2** Thickness and swelling ratio of ion-exchange sponge composites under varying CEM resin loadings (5, 10, and 20 wt%). Thickness was measured in both dry and wet states, and swelling ratios were calculated as the ratio of wet to dry thickness.

| **CEM resin loading (wt %)** | **5** | | **10** | | **20** | |
| --- | --- | --- | --- | --- | --- | --- |
| Condition | Dry | Wet | Dry | Wet | Dry | Wet |
| Thickness (mm) | 1.99±0.12 | 3.33±0.32 | 1.52±0.21 | 2.43±0.35 | 1.33±0.24 | 1.93±0.37 |
| Swelling ratio (%) | ~1.67 | | ~1.59 | | ~1.45 | |


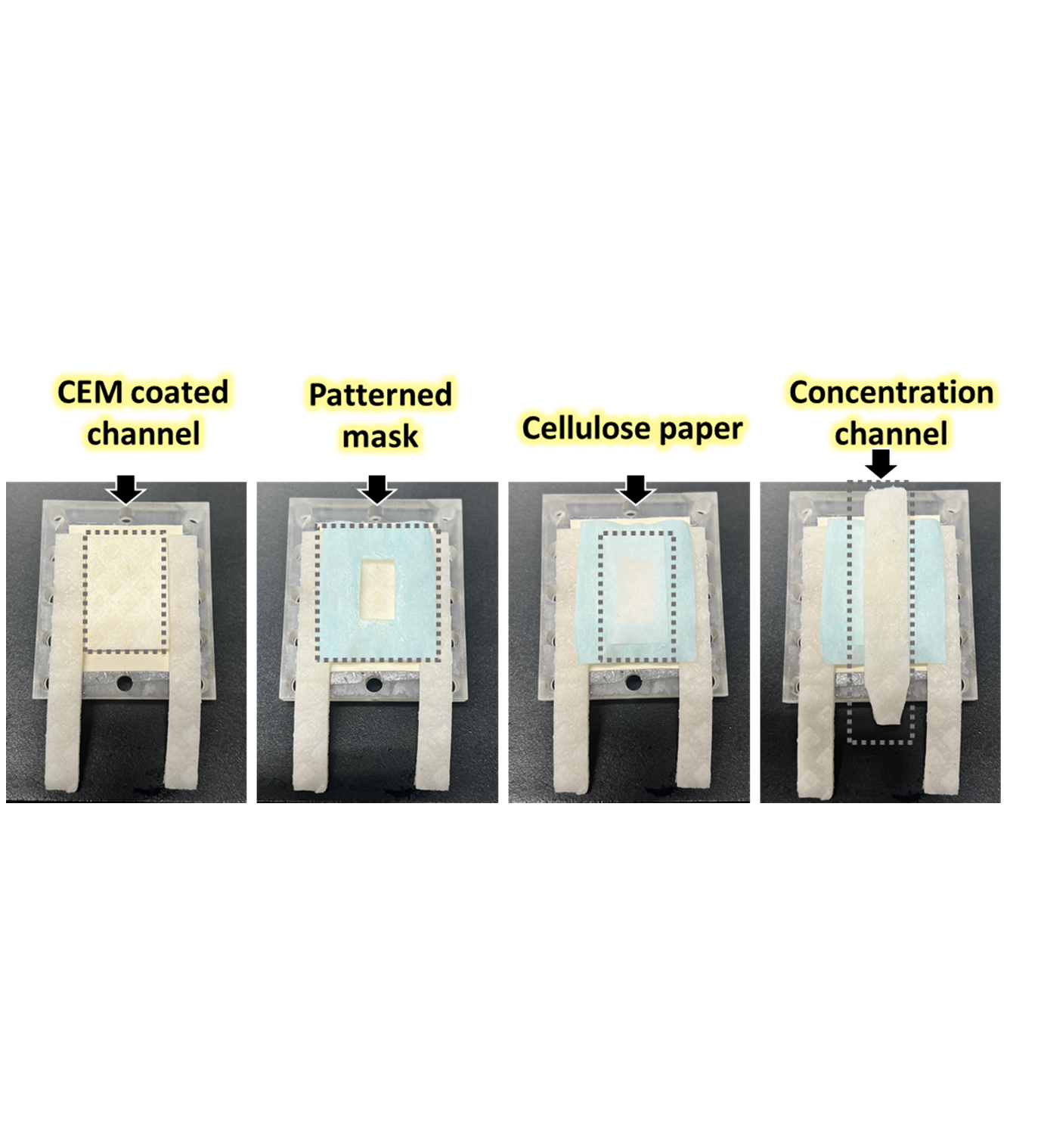


**Figure S6** assembly of the electrokinetic concentration device using cellulose-based components. (i) CEM is coated on the central region of the base channel; (ii) a patterned mask with a window is overlaid to define the active ion-selective region; (iii) cellulose paper is stacked on top to form a porous supporting matrix; (iv) a vertical pristine sponge is added as the concentration channel for diverting the waste stream.


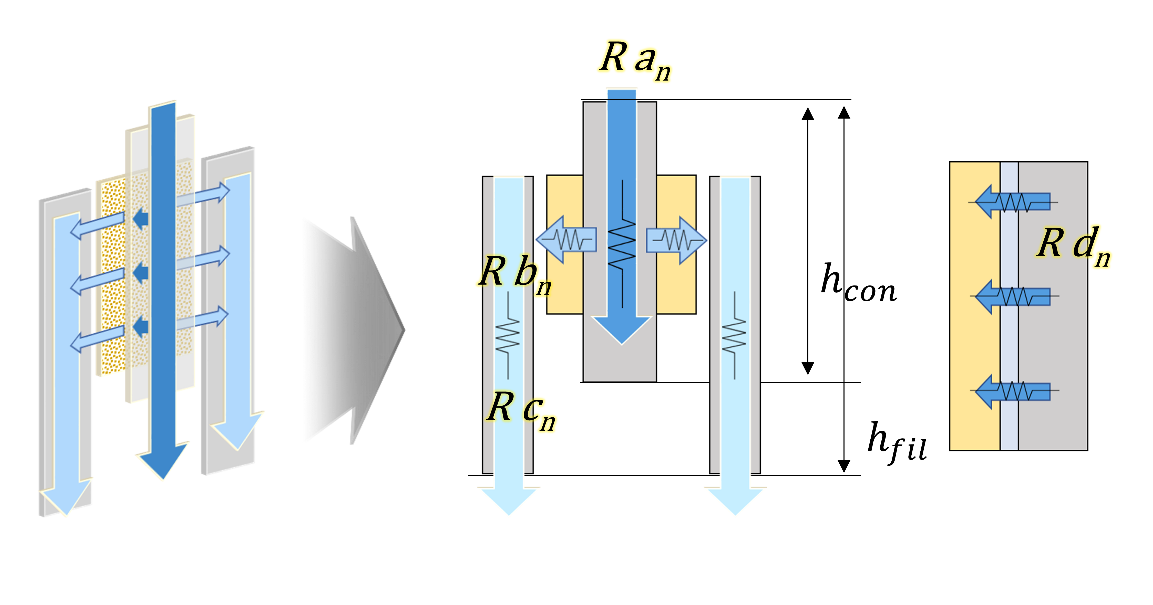


**Figure S7** Schematic illustration of water transport behavior and corresponding resistance components in a multichannel porous electrokinetic system. Water flows through multiple accessible macroscale pores (n = 1,2,3…), following distinct hydraulic pathways: the concentration channel (Ra_n_), the cation exchange membrane (Rb_n_), the filtrate channel (Rc_n_), and entry into the CEM interface (Rd_n_).

The permeability Reynolds number remains below unity, indicating that viscous forces dominate while inertial effects are negligible. Under these conditions, Darcy’s law is valid, and the flow behavior can be accurately described by a linear relationship between the pressure gradient and fluid velocity:

q = (K₁P₁ + K₂P₂ + K₃P₃ + ... + KₙPₙ) = K_t_P_t_

the total flow rate q follows a linear combination of Darcy flow under low Reynolds number conditions. Hydraulic conductivity is defined as K = kA / μL = 1 / R, where k is the permeability of the material, A is the cross-sectional area, μ is the dynamic viscosity, and L is the length of the flow path. The hydraulic resistance is thus expressed as R = μL / kA.

Flow resistance components:

- Ra_n_: flow resistance through the concentration channel

- Rb_n_: flow resistance through the Multiscale porous cation exchange membrane (MP-CEM)

- Rc_n_: flow resistance through the filtrate channel

- Rd_n_: resistance encountered when entering the MP-CEM interface

Here, n denotes the index of each water-accessible pore or flow channel (n = 1, 2, 3, …).

Because the permeability Reynolds number remains below unity, the system obeys Darcy's law, enabling linear control of flow distribution via pressure difference (Δh = h_filt_ − h_con_).

**
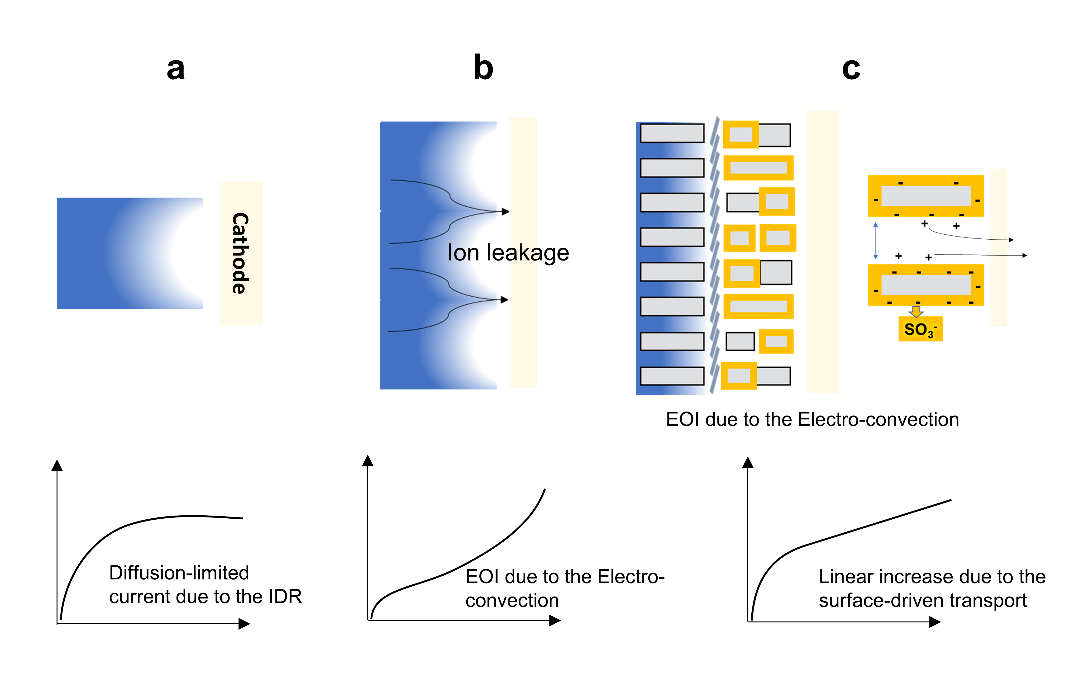
**

**Figure S8** Schematic representation of ion transport mechanisms and the corresponding current–voltage (I–V) behavior under an applied electric field. (a) In a conventional microchannel system, an ion depletion region forms near the ion-selective interface without the development of electro-convective flows, resulting in a limiting current plateau. (b) In large-scale channels (>1mm), electro-convection induces electro-osmotic instability (EOI), leading to strong ion leakage and a increase in current beyond the limiting regime (c) In multiscale porous ion-exchange membranes containing fixed sulfonate groups (SO₃⁻), a linear overlimiting current arises from ion migration along charged microscale porous surfaces, enabling continuous current increase with voltage beyond the classical diffusion limit.

The current–voltage behavior is described by two regimes. At low voltages, ion transport follows the limiting current relation:

I = I_lim_ [1 − exp(−zeV / k_B_T)]

where I_lim_ is the limiting current, z is the ion valence, e is the elementary charge, V is the applied voltage, k_B_ is the Boltzmann constant, and T is temperature.

At higher voltages, the overlimiting current (OLC) regime becomes dominant due to secondary transport mechanisms (e.g., electro-osmotic instability or surface conduction), resulting in an additional linear term:

I = I_lim_ [1 − exp(−zeV / k_B_T)] + I_surf_

The linear term I_surf_ reflects the contribution of surface conduction, where ions migrate along the charged surfaces of the multiscale porous channel, forming ion transport pathway that exceeds the classical limited current.


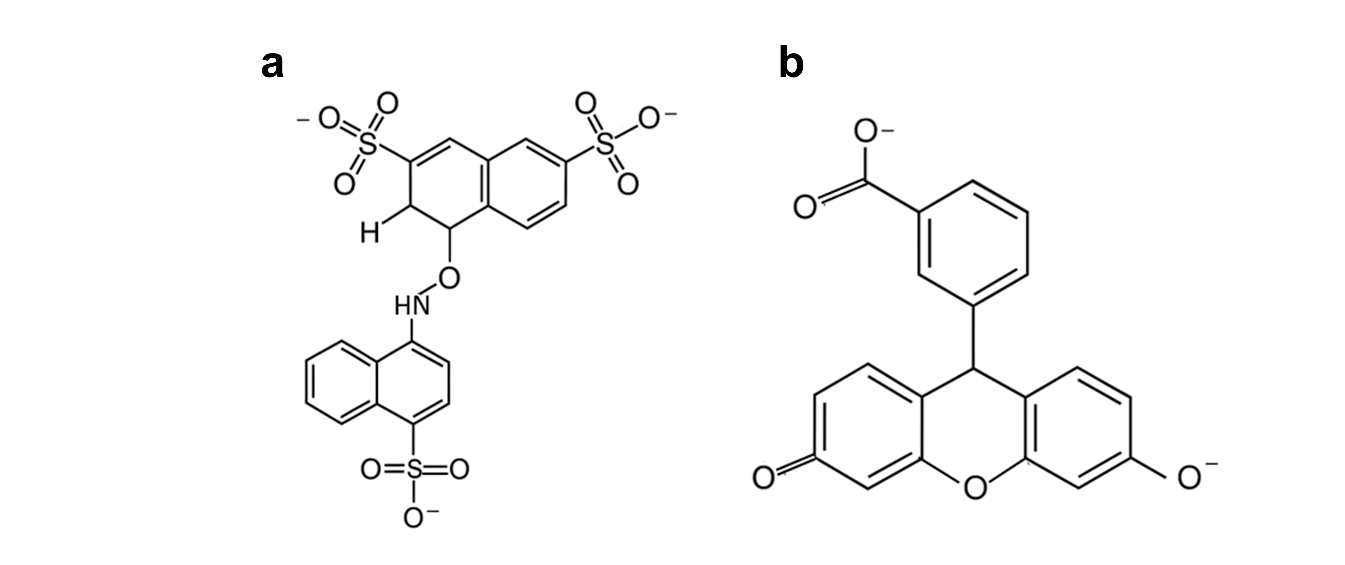


| Dyes | M_w_(g mol^-1^) | Charge pattern | λmax (nm) |
| --- | --- | --- | --- |
| Amaranth Red | 604.47 | Anionic -3 | 520 |
| fluorescein sodium salt | 376.27 | Anionic -2 | 494-495 |

**Figure S9** Properties and structure of four kinds of dyes.

**
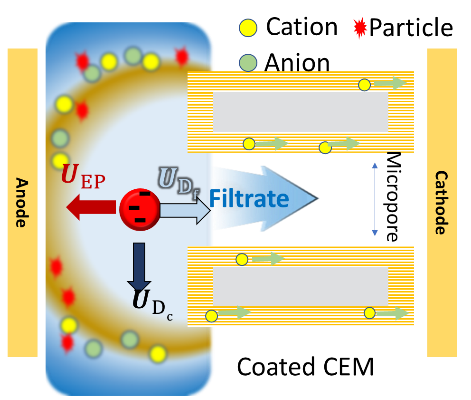
**

**Figure S10** Schematic illustration particle transport through a multichannel porous medium under electrockinetic-driven flow. Negatively charged particles are subject to competing forces: electrophoretic velocity toward the anode and drag velocity from bulk fluid flow toward the cathode (U_Drag_).

**Electrophoretic velocity of charged particles in a viscous fluid**

The steady-state electrophoretic velocity of a charged particle in a viscous fluid is given by the Smoluchowski equation ^[3]^:

U_EP_ = (ε₀ ε_r_ ζ E) / η

where ε₀ is the vacuum permittivity, ε_r_ is the fluid’s relative permittivity, ζ is the zeta potential, E is the electric field, and η is the dynamic viscosity. This relation assumes a thin electric double layer and applies to most colloids in aqueous systems. The velocity is directly proportional to both the electric field and surface potential, and inversely proportional to viscosity.

**Characteristic relaxation time of a spherical particle in a viscous fluid**

To analyze the transient behavior of a spherical microparticle (radius a) in a viscous fluid (dynamic viscosity η), we consider the particle’s motion under drag force induced by fluid flow F_Drag_. The sphere is initially at rest and experiences a viscous drag described by Stokes’ law. The equation of motion is given by:

(4/3)πa³ρ_sph_ (du/dt) = -6πηa·u + F_Drag_

where ρ_sph_ is the density of the sphere and u(t) is its velocity. Solving this first-order ODE under the initial condition u(0) = 0, we obtain:

u(t) = (F_Drag_ / 6πηa) [1 - exp(-t / τ_p_)], where τ_p_= (2ρ_sph_·a²) / (9η)

The exponential term governs the relaxation behavior of the system toward steady-state. The time scale τ_p_ represents the time required for the particle velocity to reach approximately 63% of its terminal value.

For particles with a ≲ 1 μm, τ_p_ is on the order of a few microseconds (~ μs), justifying the quasi-steady assumption throughout the experiment. Therefore,

U_Drag_ ≒ U_f_ (velocity of fluid) = q/A

A complete electrokinetic filtration occurs when U_EP_ > U_Drag_.

**
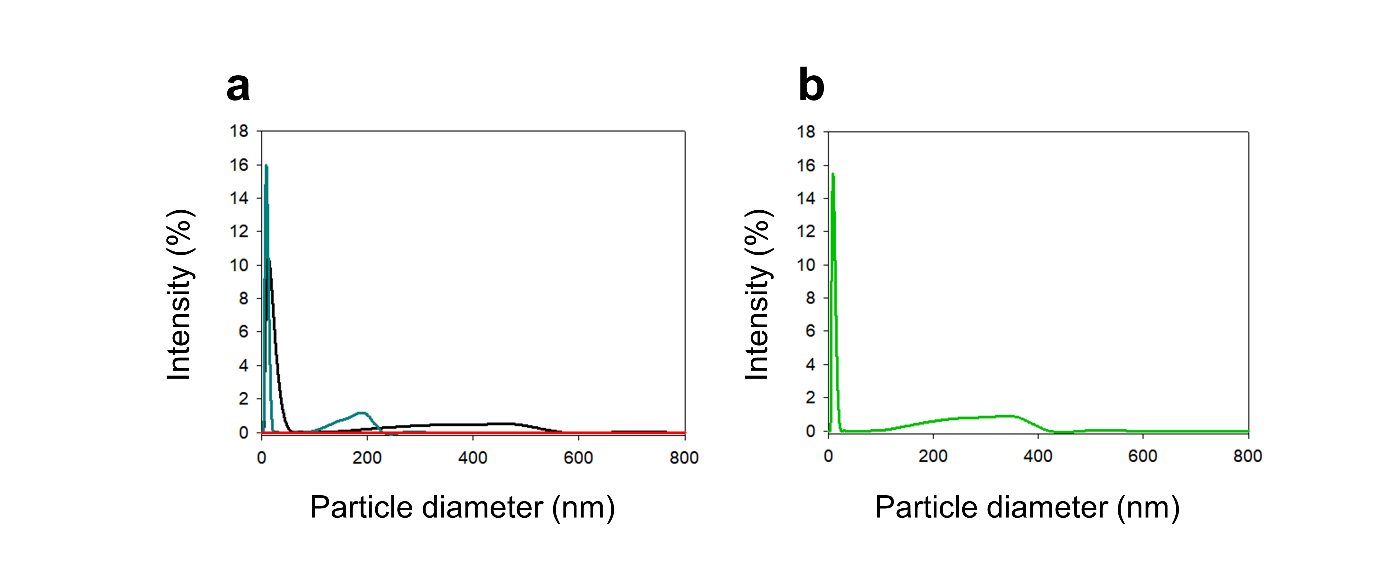
**

**Figure S11** Dynamic light scattering (DLS) analysis of particle dispersion in treated and synthetic samples. (a) DLS spectra of individual polymeric nanoparticles—polypropylene (PP, cyan) and polyethylene (PE, black)—demonstrate distinct size distributions, whereas the treated water (red) shows no measurable particle presence, indicating effective removal. (b) DLS spectrum of a mixed nanoparticle solution (PP, PE, and polystyrene (PS)) reveals the formation of a stable suspension (SS), with broad size distribution across the submicron regime, confirming the presence of well-dispersed nanoparticles.

**
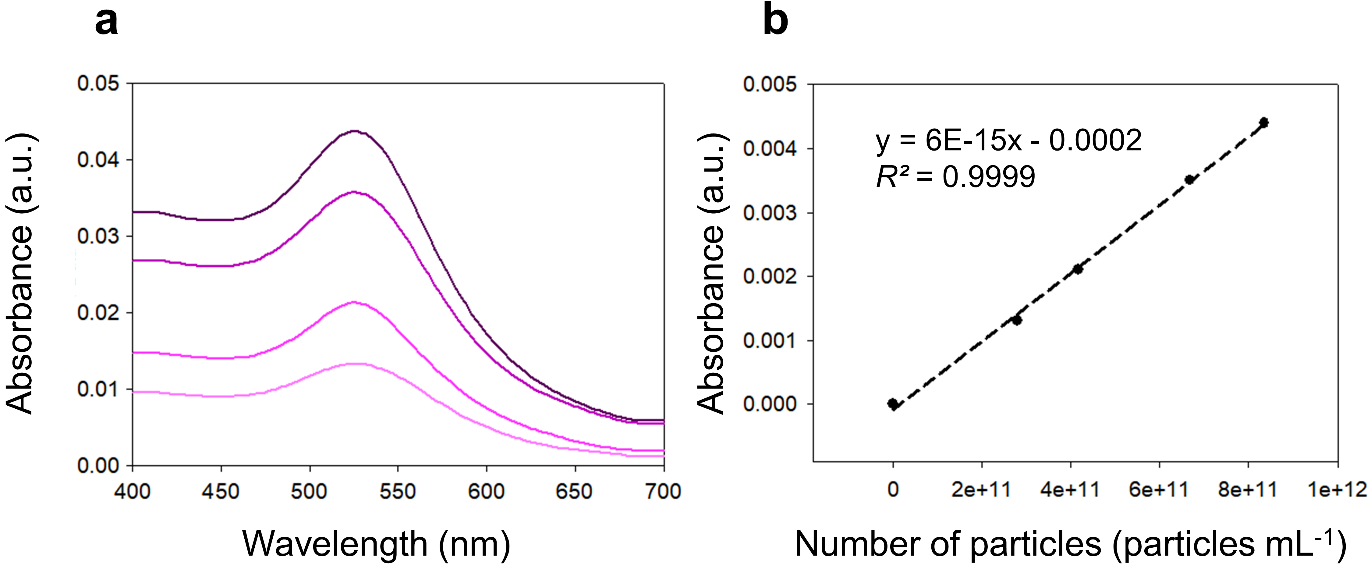
**

**Figure S12** UV–vis absorbance spectra and calibration curve of gold nanoparticle (AuNP) suspensions. (a) UV–vis spectra of AuNP feed solution and its serial dilutions (80 %, 50 %, and 33 %) show a characteristic surface plasmon resonance peak near 525 nm with decreasing absorbance corresponding to dilution. (b) Corresponding calibration curve confirms a strong linear correlation between AuNP concentration and absorbance (R² = 0.9999), validating the use of absorbance intensity for quantification of AuNPs in solution.

**Table S3.** Comparison of reported membrane nano- and ultrafiltration methods with our work.

| Membrane materials | Method | Pollutant | MPs size (nm) | Removal efficiency (%) | Flux | Ref. |
| --- | --- | --- | --- | --- | --- | --- |
| Polycarbonate (Commercial) | Size exclusion  > size of NP < size of NP (electrostatic effect) | TiO_2,_ AuNP | 10–50 | 99.9 | 150 L m^-2^h^-1^  (8kPa) | 4 |
| Polyethersulfone UF membranes (Commercial) |  | PS | 100 | 97.3 | 116 L m^-2^h^-1^  (15kPa) | 5 |
| [Ultrafiltration](https://www.sciencedirect.com/topics/engineering/ultrafiltration)  poly(sulfone) membrane |  | PE | 13–690 | - | ~130 L m^-2^h^-1^  (100kPa) | 6 |
| Bio-based nanofiber hydrogel filter |  | TiO_2,_ AuNP | 10–50 | 99.9 | 906 L m^-2^h^-1^  (3kPa) | 4 |
| Polyamide membrane with nanoscale turing structure |  | AuNP | 5 | 99.6 | 125 L m^-2^h^-^  (480kPa) | 7 |
| Ti_3_C_2_Tx^a)^ |  | PS | 80–1000 | 99.3 | 196.7 L m^-2^h^-1^ kPa^-1^ | 8 |
| PVDF-QA^b)^  (+electrostatic attraction) |  | PS | 107–1450 | 95.1 | 109 L m^-2^h^-^  (353 Pa) | 5 |
| PEI, PAA, and PAN^c)^ multilayer assemblies  (+electrostatic attraction) |  | PS | 5 | 89.9 | 861  (4 kPa) | 9 |
| PA@GO^d)^ membrane | Donnan and size exclusion or Electrostatic interaction | Trypan blue | < 2 nm | 99.8 | 75.5 L/(m2·h·bar (200~700kPa ) | 10 |
| loose polyamide with ZnO nanoparticles |  | Congo red  Methyl blue  Victoria blue B |  | 91-98 | 375 L m^-2^h^-1^ bar^-1^ (400 kPa) | 11 |
| Co@VMT^e)^ |  | rhodamine B |  | 99.7 | 119.5 L m^-2^h^-1^  (100 kPa) | 12 |
| CAU-10-H^f)^ |  | Congo red  Methyl blue  Alcian blue |  | 99.5 | 260.4 L m^-2^h^-1^ bar^-1^ (100 kPa) | 13 |
| Hierarchically structured  MP-CEM  (+ electric field) | 10~100 μm | PE/PS/PP  AuNPs  Amaranth Red  Fluorescein sodium salt | 5–800  < 2 nm (dye) | >99.9 | ~400 L m^-2^h^-1^  (< 1kPa) | Our work |

a) Titanium Carbide; b) Polyvinylidene fluoride-quaternary-ammonium; c) Polyethylenimine, poly(acrylic acid), and polyacrylonitrile; d) Polyamide and graphene oxide; e) Cobalt-functionalized vermiculite membrane;

f) synthesized aluminum-based metal-organic framework.

**
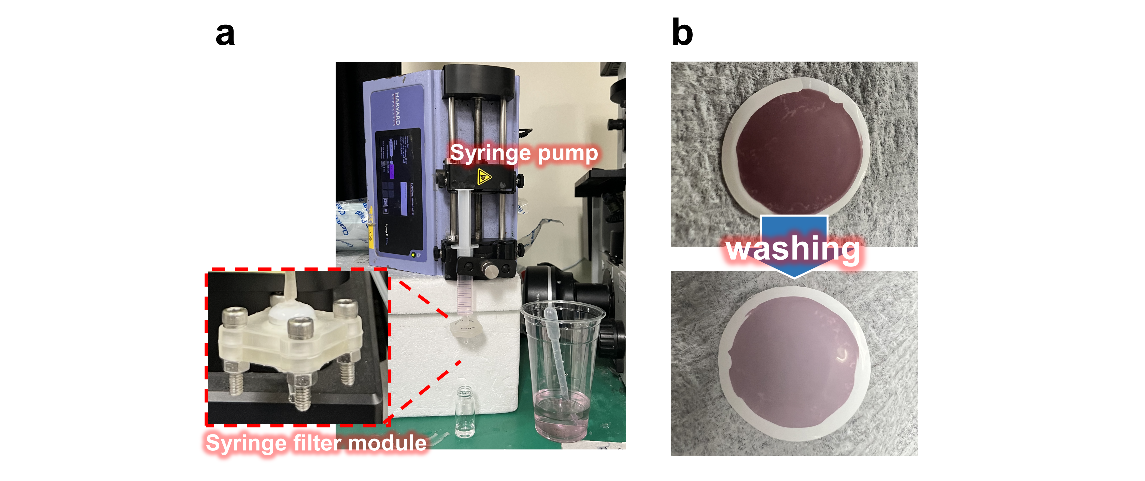
**

**Figure S13** Filtration setup and membrane appearance after AuNP filtration using a conventional 50 nm pore syringe filter. (a) Experimental setup consisting of a syringe pump and syringe filter module used to filter 5–20 nm gold nanoparticles (AuNPs) through a commercial membrane with 50 nm pore size. (b) Images of the membrane before (top) and after (bottom) washing show residual particle accumulation and irreversible fouling after use, indicating poor reusability of standard nanoporous membranes for repeated AuNP filtration.


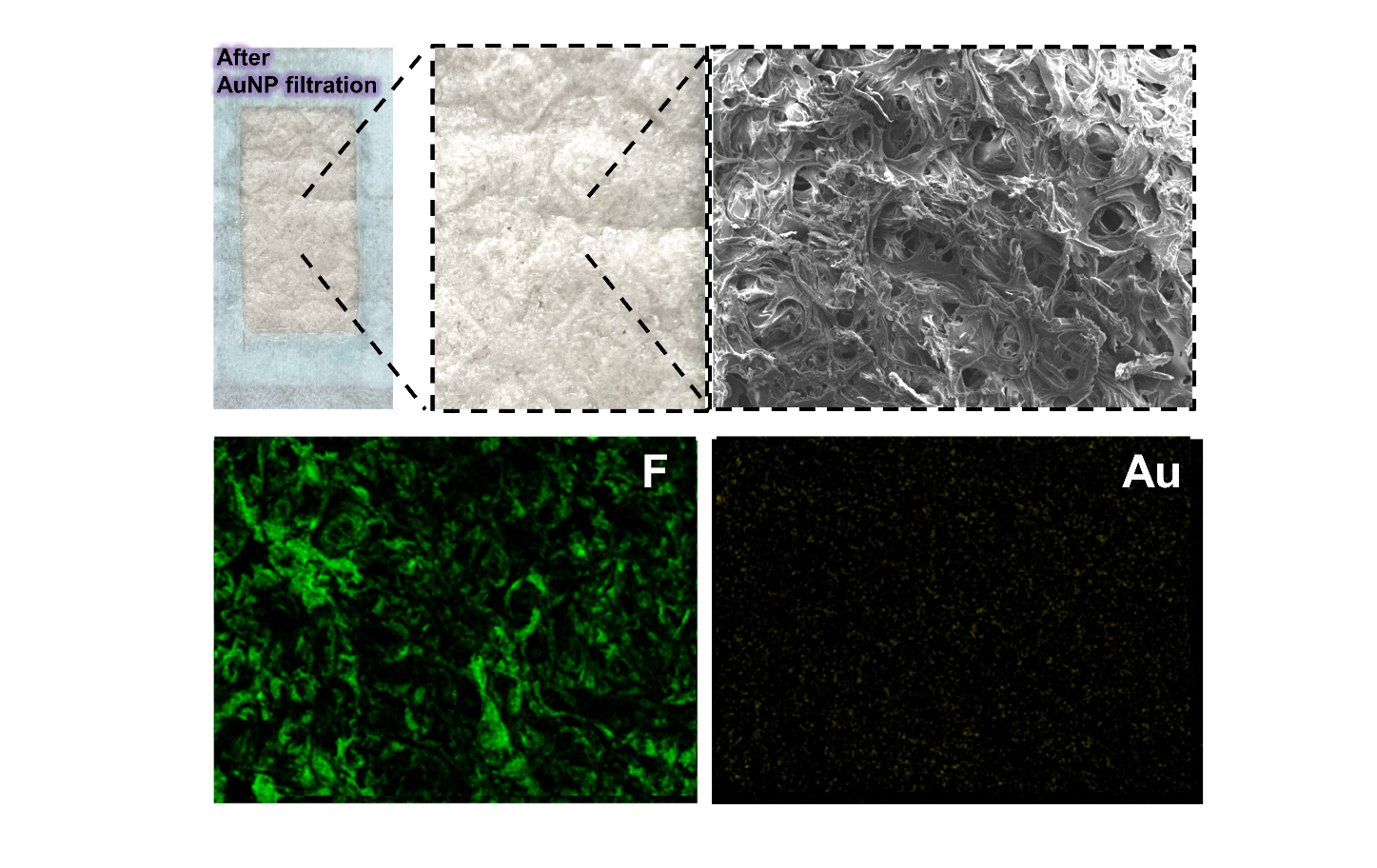


**Figure S14** Surface morphology and elemental mapping of the membrane after AuNP filtration. Optical (left), magnified (center), and SEM (right) images show the surface of the used membrane following filtration of 5–20 nm gold nanoparticles (AuNPs). Elemental mapping via EDS confirms the presence of fluorine (F, green) associated with the ion-exchange coating, while no detectable gold (Au, yellow) signal was observed. These results indicate the complete removal of AuNPs by the filtration system without residual accumulation on the membrane.

**Table S4**. Elemental composition of the sample analyzed by EDS. No detectable presence of Au was observed within the measurement limit (wt% = 0.00 ± 0.03), indicating that Au was either absent or below the detection threshold

| Element | Line Type | Wt% | Wt% Sigma | Atomic % |
| --- | --- | --- | --- | --- |
| C | K series | 41.46 | 0.09 | 50.58 |
| O | K series | 29.48 | 0.08 | 27.01 |
| F | K series | 29.06 | 0.08 | 22.41 |
| Au | M series | 0.00 | 0.03 | 0.00 |
| Total: |  | 100.00 |  | 100.00 |

**
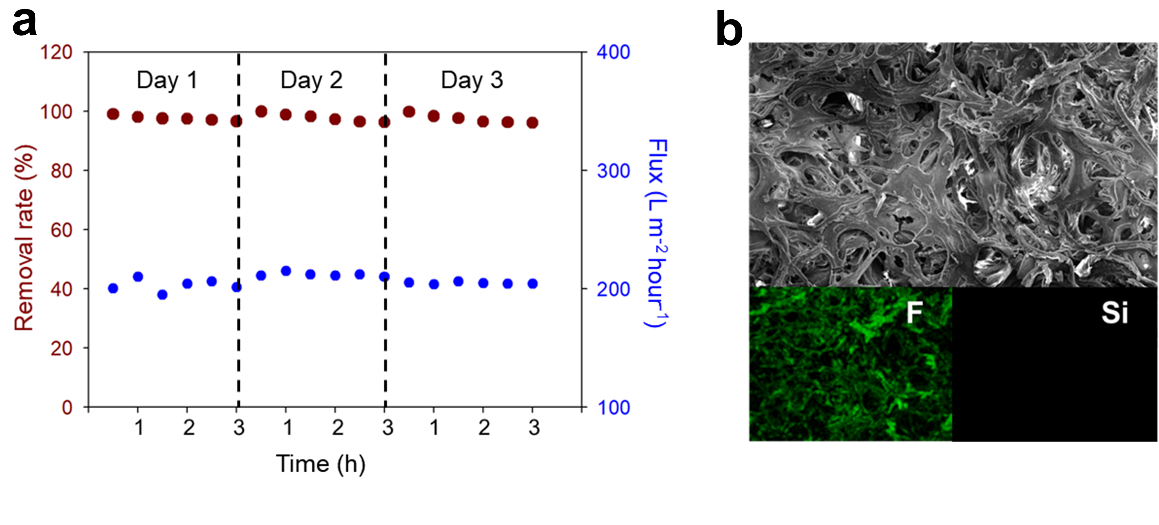
**

**Figure S15** Long-term performance evaluation of the system using a synthetic challenge solution containing humic acid (20 ppm), sodium metasilicate pentahydrate (50 ppm), CaCl₂ (177.6 mg L⁻¹), and MgSO₄·5H₂O (98.6 mg L⁻¹). (a) The removal rate of humic acid (red) gradually decreased from nearly 100% to ~96%, while the flux (blue) was maintained around 200 L m⁻² h⁻¹ over three consecutive days of operation. (b) Surface morphology and elemental mapping of the membrane after filtration. EDS confirms no detectable Si signal was observed.

**Table S5**. Elemental composition of the sample analyzed by EDS

| Element | Line Type | Wt% | Wt% Sigma | Atomic % |
| --- | --- | --- | --- | --- |
| C | K series | 44.91 | 0.10 | 53.71 |
| O | K series | 32.70 | 0.09 | 29.36 |
| F | K series | 22.39 | 0.08 | 16.93 |
| Si | M series | 0.01 | 0.01 | 0.00 |
| Total: |  | 100.00 |  | 100.00 |

**
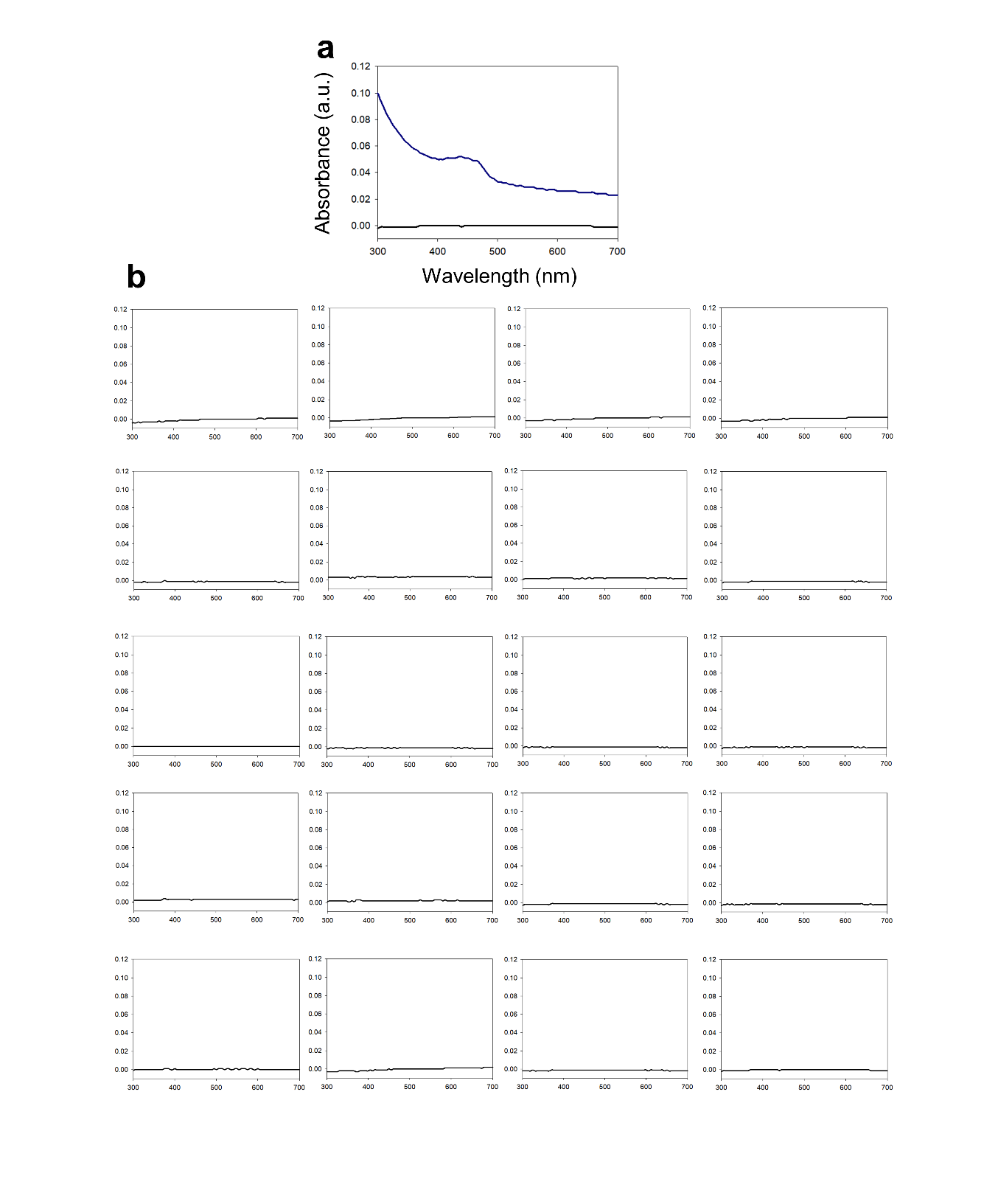
**

**Figure S16** UV–vis spectrum of (a) the mixed nanoplastic suspension and (b) the filtrates of 20 times filtration of 10 mL. Black lines represent the spectra of the corresponding treated water.

**
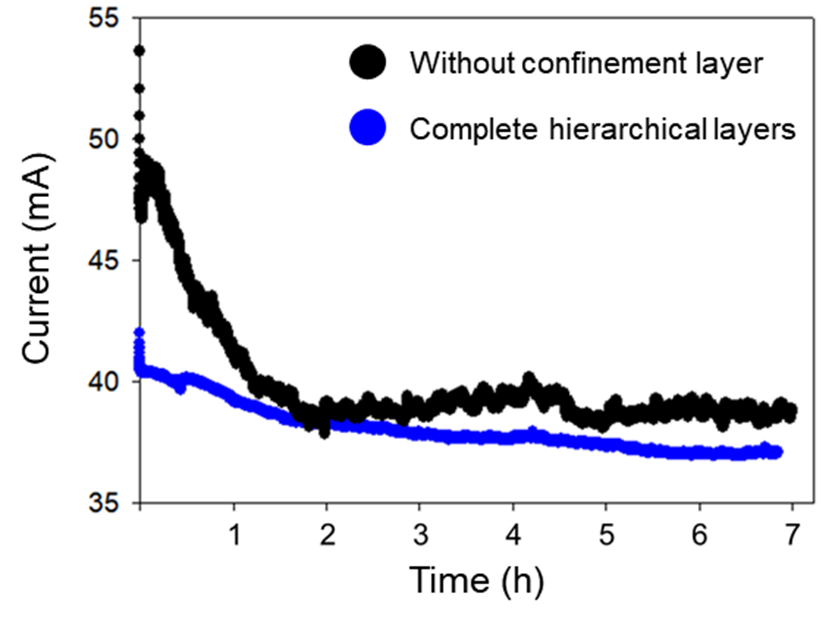
**

**Figure S17** Current responses of systems with and without a confinement layer. The complete hierarchical system (blue) shows a gradual decrease in current followed by a stable plateau with minimal fluctuations, whereas the system without a confinement layer (black) exhibits relatively unstable current behavior with pronounced fluctuations.

**Table S6.** Comparison of other electrokinetic and electrochemical filtration systems with our work.

| Channel design | Method | Pollutants | Removal efficiency (%) | Flow rate | Voltage  (V) | Cross sectional area  (width x height) | Ref. |
| --- | --- | --- | --- | --- | --- | --- | --- |
| Hierarchical porous channels | Electrokinetic | PS,PE,PP, Anionic dyes | 99.9 | > 4 ml/min | 60 | 2 x 4  cm^2^ | Our work |
| Perforated commercial CEM-microfiber channel |  | PS,PE,PP | 99.9 | ~ 1ml/min | 150 - 200 | 5x 5 x π  mm^2^ | 14 |
| Bifurcated channel |  | *E. coli* | - | 5 μL/min | 60 | 2000 x 15  μm^2^ | 15 |
| Multibranched-channel |  | PS | - | ~1 μL/min | 60 | 600 x 40  μm^2^ | 16 |
| Free flow channel |  | Anionic dyes | - | 15 μL/min | 120 | 4 x 2  μm^2^ | 17 |
| Bifurcated channel | Electrochemical | PS | 99 | < 1 μL/min | 30 | 100 x 15  μm^2^ | 18 |
| Bifurcated channel |  | PS | 99.5 | < 1 μL/min | 35 | 200 x 18  μm^2^ | 19 |

**Table S7.** Comparison of commercialized portable reverse osmosis product.

|  | Model | Power | Energy consumption | Price (accessed SEP 22, 2025) |
| --- | --- | --- | --- | --- |
| Seawater | Naked Economy  RO, Rainman | 410W | 13.67 Wh/L | $4,305 ~ 6,204 |
|  | SW150, Ampac | 326 W | 13.82 Wh/L | - |
|  | Aquifer 200, SPECTRA | 110 W | 3.49 Wh/L | $6,900.00 |
|  | VENTURA 150, SPECTRA | 108 W | 4.50 Wh/L | $7,900.00 |
| Fresh water | AquaTru Classic | 36 W | ~4.44 Wh/L | $475 |
|  | Bluevua ROPOT | 36 W | 2.3 Wh/L | $454 |
|  | RKIN ZIP | 24 W | 3.17 Wh/L | $370 |
|  | ITEHIL | 45 W | 5.33 Wh/L | $399 |
|  | Our work | ~10 W | 6 ~18 Wh/L | - |

**Comparison of the pump-driven NF/RO systems**

1. **No high-pressure pump requirement:** Unlike NF/RO units that rely on power-intensive pumps, our system operates without such components. For example, commercial portable RO devices typically require 100–400 W of continuous power [20], and even the recent compact models for freshwater still demand 36–50 W, whereas our electrokinetic platform functions effectively at <10 W. When applied to solar operation, considering ~65% effective photovoltaic output under Standard Test Conditions (STC) and ~95% charging efficiency, a 50 W solar panel used in our tests proved insufficient for stable operation. This highlights the necessity of employing larger photovoltaic modules (e.g., 100 W panels or higher) to ensure reliable continuous operation, underscoring the advantage of low-power electrokinetic systems for practical off-grid or solar-powered applications.

2. **Operation colmplexity of RO systems in remote settings:** RO systems are composed of multiple interconnected components, including pumps, filters, sensors, and control units. Operating and maintaining such systems effectively in remote areas requires technical training and specialized knowledge. However, in many rural communities or disaster-relief scenarios, such expertise and trained personnel are often lacking. As a result, even routine tasks such as filter replacement, system inspection, and troubleshooting can become challenging for general users. In practice, most freshwater RO units are used as household water purifiers.

3. **Inherent replacement demand of NF/RO membranes**: While NF/RO membranes function as consumables that progressively degrade due to fouling and scaling, our system leverages electrokinetic repulsion to actively prevent foulant deposition. This mechanism allows for repeated reuse with minimal performance decline, suggesting the potential for semi-permanent operation after further optimization.

**Table S8.** Calculation of overall system efficiency

| Component | Value | Notes |
| --- | --- | --- |
| Solar panel rating | 50 W (V_mp_ = 17.5 V, I_mp_ = 2.85 A) | Nominal |
| Effective output (based on Vmp × Imp) | 17.5 V × 2.85 A × 4 h = 199.5 Wh | Ideal (before derates) |
| PV utilization derate (65%) | 199.5 Wh × 0.65 = 129.67 Wh | Panel efficiency, wiring, temperature effects |
| Battery charging efficiency (95%) | 129.7 Wh × 0.95 = 123.17 Wh | Storage loss |
| DC–DC conversion efficiency (90%) | 123.2 Wh × 0.90 = 110.87 Wh | 12 V → 60 V step-up |
| Usable energy at load | ~110 Wh | Rounded after cumulative derates |

These figures are calculated estimates using the device specifications and realistic derating assumptions, not direct measurements.


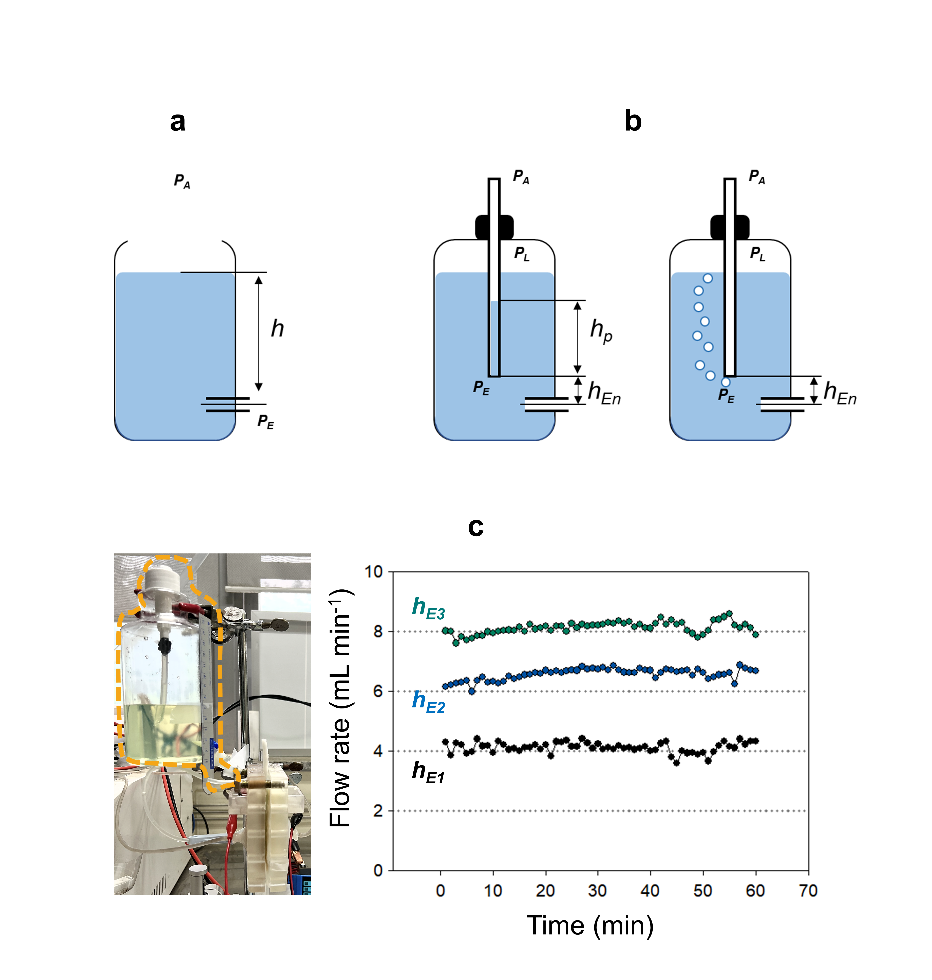


**Figure S18** Comparison of two different flow-driving mechanisms in liquid systems. **(a)** Gravity-driven flow relying on the hydrostatic head (unregulated pressure flow). **(b)** Schematic of a Mariotte bottle setup used for passive pressure regulation (constant hydrostatic pressure flow). As water is discharged through the outlet​, the internal air pressure P_L_ is balanced by air entering via the vertical tube open to the atmosphere, maintaining a constant hydrostatic head. **(b)** Stabilized water column heights h_E1​_​, h_E2​_, and h_E3​_ over time at different outlet levels, confirming constant hydrostatic head during discharge.

Unlike conventional open tanks, where the pressure head and hence flow rate decrease with the water level, the Mariotte configuration provides a constant pressure at the outlet by coupling atmospheric pressure via an air-inlet tube extending into the sealed reservoir.

The pressure at end of the vertical tube, denoted as P_E_, can be expressed as:

P_E_ = ρgh_p_ + P_L_

where

- ρ is the density of water
- g is the gravitational acceleration,
- h_p_ is the vertical distance between the water surface and the lower end of the tube,
- P_L_ is the air pressure inside the sealed space above the water,
- P_A_, the atmospheric pressure.

Initially, the internal air pressure is equal to the atmospheric pressure (P_L_ = P_A_). As water flows out, the internal air pressure P_L_ decreases, reducing h_p_. However, once h_p_ reaches the end of the pipe, air enters through the vertical tube as bubbles, thereby restoring the pressure at the outlet to P_A_ and maintaining:

P_E_ ≈ P_A_ → Flow rate remains constant.

The flow velocity v at the outlet is thus given by:

v = √(2gh_E_)

where h_E_ remains constant as long as air bubbling replenishes the internal pressure and balances the hydrostatic head.


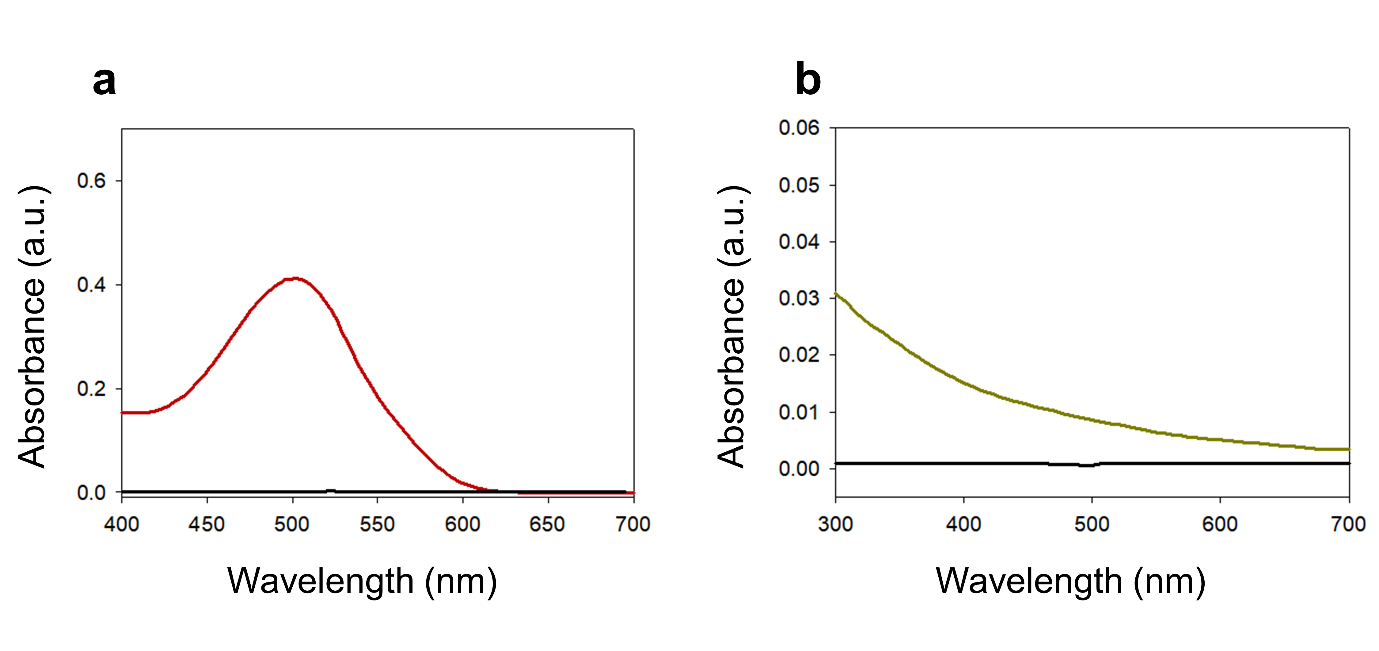


**Figure S19** UV–vis spectra of (a) aqueous dispersion of Amaranth red at a concentration of 200 mg L⁻¹, and (b) a mixed suspension of polypropylene (PP) and polyethylene (PE), each at 100 mg L⁻¹ (total 200 mg L⁻¹). Black lines represent the spectra of the corresponding treated water.


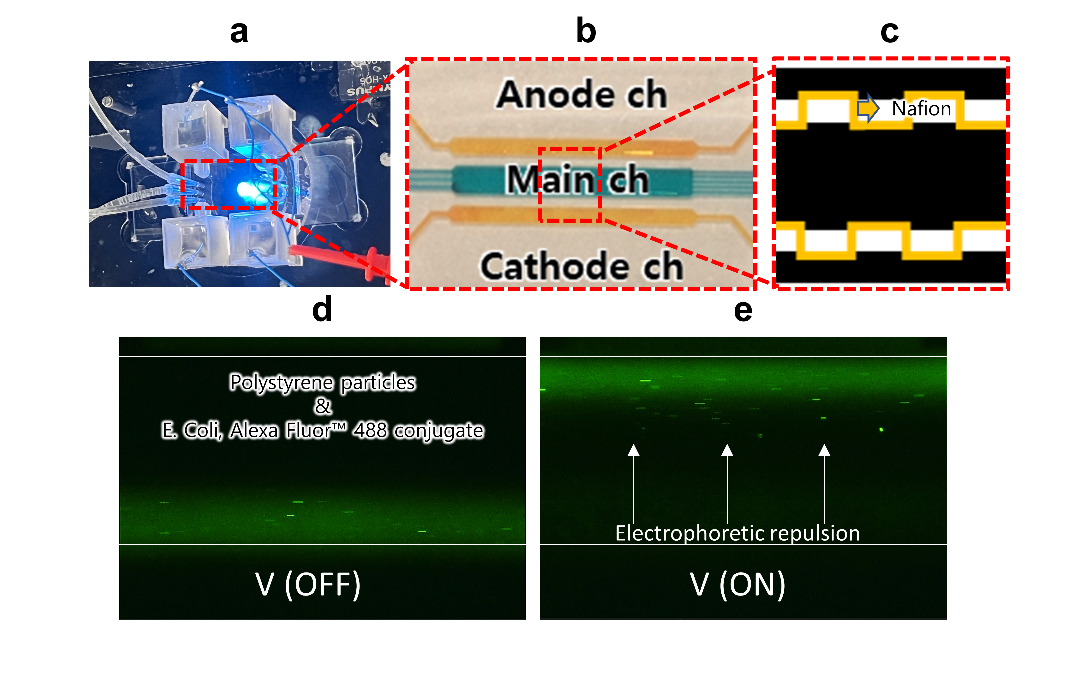


**Figure S20** (a) Photograph of the ICP chip device. (b) Schematic illustrations of the main channel (blue) and orthogonal anode/cathode channels (orange) with (c) patterned Nafion junctions (Yellow) enabling localized ion depletion. (d) Fluorescence microscopy images of a mixture of polystyrene particles and Alexa Fluor™ 488–conjugated *E. coli* in the absence and (e) presence of applied voltage, showing electrophoretic repulsion of negatively charged species under ICP.

**References**

1. Goldberg, U. C. Exploring a Three-Equation R–k–ε Turbulence Model. *Journal of Fluids Engineering*, 1996, 118, 795–799.
2. S. V. Pham, H. Kwon, B. Kim, J. K. White, G. Lim, J. Han, Phys. Rev. E 2016, 93
3. Nazockdast, E.; Morris, J. F. Pair-Particle Dynamics and Microstructure in Sheared Colloidal Suspensions:Simulation and Smoluchowski Theory. *Physics of Fluids*, 2013, 25.
4. Jiang, M.; Jing, C.; Lei, C.; Han, X.; Wu, Y.; Ling, S.; Zhang, Y.; Li, Q.; Yu, H.; Liu, S.; et al. A Bio-Based Nanofibre Hydrogel Filter for Sustainable Water Purification. *Nature Sustainability*, 2024, 7, 168–178.
5. Wan, H.; Shi, K.; Yi, Z.; Ding, P.; Zhuang, L.; Mills, R.; Bhattacharyya, D.; Xu, Z. Removal of Polystyrene Nanoplastic Beads Using Gravity-Driven Membrane Filtration: Mechanisms and Effects of Water Matrices. *Chemical Engineering Journal*, 2022, 450, 138484.
6. Enfrin, M.; Lee, J.; Le-Clech, P.; Dumée, L. F. Kinetic and Mechanistic Aspects of Ultrafiltration Membrane Fouling by Nano- and Microplastics. *Journal of Membrane Science*, 2020, 601, 117890.
7. Tan, Z.; Chen, S.; Peng, X.; Zhang, L.; Gao, C. Polyamide Membranes with Nanoscale Turing Structures for Water Purification. *Science*, 2018, 360, 518–521.
8. Yang, L.; Cao, X.; Cui, J.; Wang, Y.; Zhu, Z.; Sun, H.; Liang, W.; Li, J.; Li, A. Holey Ti3C2 Nanosheets Based Membranes for Efficient Separation and Removal of Microplastics from Water. *Journal of Colloid and Interface Science*, 2022, 617, 673–682.
9. Wang, R.; Zhang, L.; Chen, B.; Zhu, X. Low-Pressure Driven Electrospun Membrane with Tuned Surface Charge for Efficient Removal of Polystyrene Nanoplastics from Water. *Journal of Membrane Science*, 2020, 614, 118470.
10. Zhan, X.; Ge, R.; Huo, T.; Lu, J.; Li, J. Highly Permeable PA@GO Loose Nanofiltration Membranes Enabled by Hierarchical Transport Channels for Efficient Dye Removal. *Chemical Engineering Journal*, 2023, 476, 146831.
11. Liu, Y.; Mo, J.; Ding, H.; Cheng, Y.; Zhang, Z.; Wang, C.; Li, X. Ultrafast Loose Nanofiltration Membrane Intercalated by In-Situ Grown Nanoparticles for Dye Purification and Reuse. *Desalination*, 2023, 551, 116439.
12. Tian, M.; Liu, Y.; Zhang, S.; Yu, C.; Ostrikov, K.; Zhang, Z. Overcoming the Permeability-Selectivity Challenge in Water Purification Using Two-Dimensional Cobalt-Functionalized Vermiculite Membrane. *Nature Communications*, 2024, 15.
13. Hundessa, N. K.; Hu, C.-C.; Kang, D.-Y.; Chou, P.-C.; Ajebe, E. G.; Lee, K.-R.; Lai, J.-Y. Ultra-High Flux Loose Nanofiltration Membrane Based on Metal Organic Framework (CAU-10-H)/P84 Co-Polyimide for Dye/Salt Fractionation from Industrial Waste Water. *Desalination*, 2024, 586, 117871.
14. M. Lee, W. Choi and G. Lim, Electrokinetic-assisted filtration for fast and highly efficient removal of microplastics from water, *Chemical Engineering Journal*, 2023, 452, 139152.
15. R. Kwak, S. J. Kim, J. Han, Continuous-Flow Biomolecule and Cell Concentrator by Ion Concentration Polarization. *Anal. Chem*. 2011, 83, 7348–7355.
16. H. Jeon, H. Lee, K. H. Kang, G. Lim, Ion concentration polarization-based continuous separation device using electrical repulsion in the depletion region. *Sci Rep* 2013, 3.
17. V. A. Papadimitriou, L. I. Segerink, J. C. T. Eijkel, Free Flow Ion Concentration Polarization Focusing (FF-ICPF). *Anal. Chem*. 2020, 92, 4866–4874.
18. J. R. Thompson, L. M. Wilder, R. M. Crooks, Filtering and continuously separating microplastics from water using electric field gradients formed electrochemically in the absence of buffer. *Chem. Sci*. 2021, 12, 13744–13755.
19. C. D. Davies, E. Yoon, R. M. Crooks, Continuous Redirection and Separation of Microbeads by Faradaic Ion Concentration Polarization. *ChemElectroChem* 2017, 5, 877–884.
20. J. Yoon, H. J. Kwon, S. Kang, E. Brack, J. Han, Portable Seawater Desalination System for Generating Drinkable Water in Remote Locations. *Environ. Sci. Technol*. 2022, 56, 6733–6743.
